# Integrated widely-targeted metabolome and transcriptome analysis provide novel insights into the regulation of nutritional formation in *Artocarpus nanchuanensis* fruit

**DOI:** 10.3389/fpls.2026.1800089

**Published:** 2026-04-13

**Authors:** Shumin Wang, Kun Zhou, Tao Guo, Zhiruo Zhang, Hong Yang, Junping Quan, Wanpeng Xi

**Affiliations:** 1 College of Horticulture and Landscape Architecture, Southwest University, Chongqing, China; 2 Nanshan Botanical Garden Management Office, Chongqing, China; 3 Dima Holdings Co.,Ltd., Chongqing, China; 4 Chongqing Municipal Agricultural School, Chongqing, China

**Keywords:** *Artocarpus nanchuanensis* fruit, nutritional assessment, metabolomics profiling, transcriptomic analysis, flavonoids

## Abstract

*Artocarpus nanchuanensis* is a critically endangered tree endemic to Chongqing, China. Although a large number of seedlings have been successfully cultivated and preserved, their overall utilization rate remains suboptimal. The nutritional formation of fruit remains elusive. Here, we analyzed three fruit stages (green mature, color-breaking, complete ripeness), to gain more insight through transcriptomics and widely targeted metabolomics. 1,662 metabolites were identified, with flavonoids being the most abundant (18.65%). Alkaloids accounted for 7.89%. The complete ripeness fruits were rich in nepetin, luteolin, pterolactam, tetrahydroharmol, saccharides, organic acids, amino acids, and vitamins. Vitamin C content was highest in green mature fruit (570.06 μg/g) and decreased slightly with ripening (542.16 μg/g in complete ripeness fruit). Diterpene alkaloids Anthriscifolcine A has not yet been reported in other Moraceae plants. Most flavonoids reached their peak concentrations at the color-breaking stage, suggesting this may be the optimal period for their industrial extraction. Some MYB, ERF, WRKY transcription factors potentially regulated the synthesis of flavonoids, alkaloids and other substances. 264 DEGs, enriched in the plant hormone transduction pathway, regulated the ripening of fruits. The constructed flavonoids and saccharides metabolic pathway unveiled the regulatory networks of nutritional components in *A. nanchuanensis* fruits. These findings offer valuable insights that can inform future nutritional research and advance medicinal development.

## 1 Introduction

With the rapid development of modern science and technology, people’s demands for the nutritional value and health benefits of food are gradually increasing. As an indispensable source of food in human daily diet, plant fruits are not only favored for their rich taste and flavors, but also for their positive impact on human health from various nutrients. *Artocarpus nanchuanensis* stands out for its unique flavor and nutritional value, and is one of the potential research directions for the development of fruit product.

*A. nanchuanensis* is a perennial evergreen tree unique to the Chongqing of China. It is a rare and endangered plant of the Moraceae (He et al., 2022). *A. nanchuanensis* has high nutritional and medicinal value, and is an ideal tree species for cultivating and developing new fruit industries. It is being introduced and cultivated in many tropical regions. The fruit of *A. nanchuanensis* contains various nutrients that are beneficial to human health, such as amino acids, vitamins, trace elements, polysaccharides, proteins, esters, etc. Moreover, the health benefits included preventing cardiovascular diseases, regulating the gastrointestinal tract, and maintaining physiological functions such as prevention and treatment of skin allergies, oxidation, and aging (Ren et al., 2013).

Fruit ripening is a complex physiological process accompanied by complex physiological and biochemical changes. Exploring the formation and changes of various substances during fruit ripening would be helpful for the elucidation and improvement of nutritional value and flavor (Lu et al., 2022). During the development of fruit, nutrients such as carbohydrates, proteins, vitamins, etc. undergo dynamic changes. At the same time, metabolic substances that reflect the flavor and color of the fruit (such as organic acids, carotenoids, anthocyanins) undergo regular changes. Elaboration of these characteristics is of great significance for improving fruit quality in the future (Wang et al., 2022).

Flavonoids are water-soluble secondary metabolites present in plants and classified into six types, i.e., chalcones, flavones, flavonols, flavandiols, anthocyanins, and condensed tannins (Liu et al., 2021). Flavonoids play crucial roles in flower coloration, pollen germination, pollination and seed dispersal, UV filtration, pathogen resistance, shade tolerance, and so on (Guo et al., 2024; Mo et al., 2024). In addition, flavonoids are the main ingredients of traditional Chinese medicines because their anti-inflammatory, antibacterial, antioxidant, and anti-tumor (Ma et al., 2024). In recent years, with the in-depth study of the biosynthesis pathways of secondary metabolites in plants, researchers have not only cloned some key enzyme genes in the phenylpropanoid-flavonoid pathways (chalcone synthase: CHS; chalcone isomerase: CHI; phenylalanine ammonia-lyase: PAL; dihydroflavonol 4-reductase: DFR; flavonol synthase: FLS; etc.), but also conducted functional studies on genes regulating the expression of key enzymes using model plants (Jiang L, et al., 2023) 23 sugars were screened from the pulp of the Korla fragrant pear fruits, and D- lactose was significantly different in fruits of different appearances (Jiang W, et al., 2023). So far, the metabolic synthesis pathways and related functional genes of flavonoids have been confirmed and systematically studied, making the flavonoid metabolic synthesis pathway one of the most extensively studied metabolic systems in plant secondary metabolism (Martínez-Rivas et al., 2023).

Alkaloids are a class of alkaline nitrogen-containing organic compounds widely present in plants and characterized with broad prospects in pharmacy, such as isoquinoline, quinolone, plumerane, pyrrole, piperidine alkaloids, etc (Bhambhani et al., 2021). Because alkaloids are mostly alkaline and could form salts with acids, harboring antioxidant, anti-diabetes, anti-inflammatory and anti-hypertensive pharmacological activities (Zhu et al., 2023). Although alkaloids in plants have shown potential therapeutic effects in medicine, there are still many problems that need to be overcome, such as unclear mechanisms and uncertain specific active ingredients. In addition, some compounds may have side effects or potential risks of abuse, therefore further research is needed to evaluate the types and safety of alkaloids in plants.

As a high-quality germplasm resource that combines medicinal, edible, timber, and ornamental values, *A. nanchuanensis* has broad application prospects and important protection and development value. Therefore, based on the study of the nutritional function of *A. nanchuanensis*, it is necessary to analyze the spatiotemporal changes of its fruit nutrients. This research first used metabolomics methods to construct the metabolic profile of fruits from different developmental stages of *A. nanchuanensis* plants. At the same time, based on the clarification of the main nutrients in the fruit of *A. nanchuanensis*, combined analysis and functional prediction were conducted using metabolome and transcriptome data to reveal the molecular basis of the formation and changes in the nutritional value of *A. nanchuanensis*. Our results will provide theoretical basis and technical guidance for the development and utilization of the nutritional value of *A. nanchuanensis*, further promoting the development of *A. nanchuanensis* industry.

## 2 Materials and methods

### 2.1 Plant materials

For this study, *A. nanchuanensis* fruit samples were collected from a natural population located in Qijiang (28° 45’ 54.581” N, 106° 41’ 1.828” E). All fresh fruits at three developmental stages (green mature, color-breaking, and complete ripeness) were collected from 15-year-old trees (artificially propagated, cultivated trees). For each stage, nine fruits were randomly selected from three trees (three fruits per tree). These nine fruits were then cut into small pieces, pooled together, and thoroughly mixed to create a representative bulk sample. From this pooled sample, three biological replicates were taken, each consisting of an aliquot of the mixed tissue. The replicates were immediately flash-frozen in liquid nitrogen and stored at -80 °C until further use.

### 2.2 Measurement of quality evaluation indicators

Fruit color values were determined using a compact lab colorimeter (CR-400/CR-41, Konica Minolta, Tokyo, Japan), where L* indicates brightness (0-100), a* represents the green-to-red spectrum (-60 to 60), and b* reflects the blue-to-yellow spectrum (-60 to 60). The titratable acid (TA) and total soluble solid (TSS) were quantified with a digital refractometer (PAL-BX/ACID12, Atago, Tokyo, Japan). Vitamin C content was detected through Ascorbic acid (AsA) content assay kit (Solarbio Life Sciences, Beijing, China) according to the instructions. All tests were performed in triplicate.

### 2.3 Transcriptomic profiling and bioinformatics analysis

Total mRNA was extracted from nine fruit samples (three stages × three replicates) using the Mag-Bind Tissue Direct mRNA Kit (OMEGA, USA). RNA quality (integrity, concentration and purity) was verified through 1% agarose gel electrophoresis, Qsep400 bioanalyzer, Qubit 4.0 Fluorometer, and NanoPhotometer spectrophotometer. Fragmented mRNA served as a template for cDNA synthesis, followed by purified, end repair, adapter ligation, and PCR enrichment to construct sequencing libraries (NEBNext^®^ UltraTM RNA Library Prep Kit, Illumina, USA). Libraries (FGT: fruit-L (green mature fruit); FCT: fruit-C (color-breaking fruit); FMT: fruit-S (complete ripeness fruit)) were quantified by Q-PCR (the concentration >2 nM) and sequenced on an Illumina platform (raw data deposited at NGDC, PRJCA033058). Nine sequencing libraries with three biological replicates each were generated. The sequencing yielded 65.93 Gb of high-quality clean data, with 93.16-93.87% of bases scoring Q30 and GC content ranging from 46.68% to 48.02%. (**Supplementary Figure 1**). Approximately 90.02-96.10% of clean reads uniquely mapped to the *A. nanchuanensis* reference genome (BioProject: PRJNA624965).

Gene annotation was performed by aligning sequences against KEGG, GO, NR, Swiss-Prot, trEMBL, and KOG databases (E-value ≤1e-5). Gene expression levels were quantified with featureCounts to generate gene alignment statistics. Following this, FPKM (Fragments Per Kilobase Million) values for each gene were derived using gene length. FPKM remains the most widely adopted method for estimating gene expression levels.

Transcription factors (TFs) were predicted using iTAK software, which integrates two databases, PlnTFDB and PlantTFDB (Jin et al., 2014). Differential gene expression analysis between the two groups was conducted using DESeq2, with significant differentially expressed genes (DEGs) defined by an adjusted P-value (FDR) < 0.05 and an absolute log_2_ fold change ≥ 1. The Benjamini-Hochberg method was applied for multiple testing correction. Enrichment analysis was subsequently performed using the hypergeometric test, examining KEGG pathways and GO terms separately based on the identified DEGs.

The weighted geneco-expression network analysis (WGCNA, V1.71) constructs a hierarchical clustering tree based on pairwise correlations of gene expression profiles and identifies co-expression modules. In the figure, each color represents a distinct module, with genes of the same color on the clustering tree belonging to the same functional module. The minimum module size was set to 50. The merge cut height was set at 0.25.

### 2.4 Metabolomic profiling and pathway analysis

Metabolite dynamics during fruit development were assessed via broadly targeted metabolic analysis based on UPLC-MS/MS (Metware Biotechnology Co., China). The samples were subjected to vacuum freeze-drying in a Scientz-100F lyophilizer and subsequently ground into a homogeneous powder. Freeze-dried samples (50 mg) were extracted with 70% methanol (-20 °C) and analyzed on an Agilent SB-C18 column (1.8 µm, 2.1 × 100 mm) using a water-acetonitrile gradient with 0.1% formic acid. Multiple reaction monitoring (MRM) scanning is employed for the quantitative analysis of target substances. This method utilizes five key parameters, declustering potential (DP), collision energy (CE), retention time (RT), precursor ion (Q1), and product ion (Q3), to detect and measure the relative abundance of substances across different samples, thereby generating both qualitative and quantitative data.

Metabolites in the samples were identified, which mainly relies on a self-constructed database (Metware database) based on the retention time, precursor ions, and product ions of authentic standards, combined with the comparison of characteristic peak areas. The detection results reflect the intensity variation trends of these markers among different sample groups, rather than absolute concentration values determined using independent standard curves. Analyst 1.6.3 and MultiQuant software were used to analyze data. Following quality control (QC) analysis, data stability was assessed by examining the proportion of metabolites with coefficient of variation (CV) values below a predefined threshold, using the empirical cumulative distribution function (ECDF). Unsupervised principal component analysis (PCA) was then performed using the prcomp function in R (www.r-project.org), with data subjected to unit variance scaling prior to analysis.

FGM, FCM and FMM represent the metabolome data corresponding to green mature, color-breaking, and complete ripeness fruits, respectively. For two-group comparative analysis, differentially accumulated metabolites (DAMs) were decided by VIP (VIP > 1) and Log_2_FC (|Log_2_FC| ≥ 1.0). VIP values were extracted from OPLS-DA result, which also contain score plots and permutation plots, was generated using R package MetaboAnalystR. DAMs were annotated to KEGG pathways, through KEGG compound database (http://www.kegg.jp/kegg/compound/) and KEGG Pathway database (http://www.kegg.jp/kegg/pathway.html). Enrichment analysis employed MSEA with a hypergeometric test’s p-values (p <0.05).

### 2.5 Integrated analysis of plant transcriptome and widely targeted metabolome

Integrating multi-omics data for analysis can systematically and comprehensively elucidate the functions and regulatory mechanisms of biomolecules, and screen genes and metabolites in key metabolic pathways for further in-depth experimental analysis and applications. The transcriptome data FGT of green mature fruits was analyzed jointly with the metabolome data FGM, the transcriptome data FCT of color-breaking fruits was analyzed jointly with the metabolome data FCM, and the transcriptome data FMT of complete ripeness fruits was analyzed jointly with the metabolome data FMM. KEGG pathway enrichment analysis and expression correlation analysis were performed for genes and metabolites. The correlation method used the cor function in R to calculate the Pearson correlation coefficient between genes and metabolites. Correlation results with an absolute correlation coefficient greater than 0.8 and p-value less than 0.05 were selected. A positive correlation coefficient indicated a positive correlation between genes and metabolites, while a negative value indicated a negative correlation.

## 3 Results

### 3.1 Changes of physical properties and flavor indicators during fruit ripening

**Figure 1:**
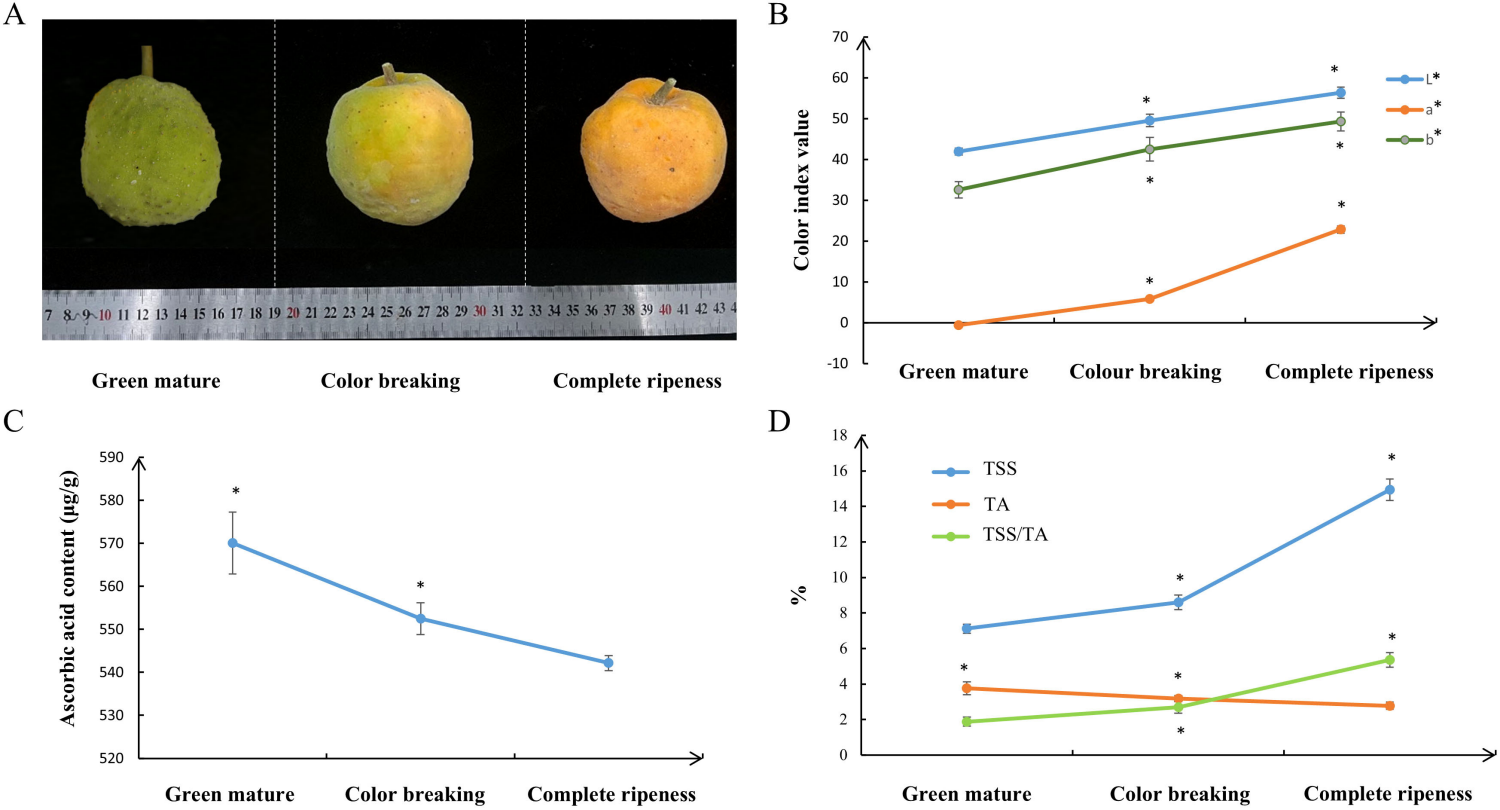
Phenotypic and flavor characteristics of *A. nanchuanensis* fruits at three developmental stages. **(A)** Representative fruits of *A. nanchuanensis* at three distinct ripening stages, ordered from left to right: green mature, color-breaking, and complete ripeness. **(B)** Colorimetric analysis of fruits at three developmental stages, including the parameters L*, a*, b*. Data represent mean values ± SD (n=9). *denotes statistically significant differences at p < 0.05. **(C)** Quantification of ascorbic acid (vitamin C) content during fruit maturation. Data represent mean values ± SD (n=3). *denotes statistically significant differences at p < 0.05. **(D)** Comparative analysis of TSS, TA, and TSS/TA ratio across the three stages. Data represent mean values ± SD (n=9). *denotes statistically significant differences at p < 0.05. Independent t-tests demonstrated that there was significant difference (P<0.05).

The fruits of *A. nanchuanensis* were analyzed at three ripening stages: green mature, color-breaking, and complete ripeness. As shown in the **Figure 1A** (from left to right), the color parameters varied significantly (**Figure 1B**): L* values (lightness) decreased with ripening (56.3 for green mature, 49.5 for color-breaking, and 41.9 for complete ripeness), while a* (green-red axis) shifted from 22.8 (green mature) to -0.62 (complete ripeness), indicating a loss of green pigmentation. The b* values (yellowness) also declined from 49.3 (green mature) to 32.5 (complete ripeness). Flavor-related traits also changed during ripening. Total soluble solids (TSS) increased progressively from 7.12 in green mature fruit to 14.95 in complete ripeness fruit (**Figure 1D**). In contrast, titratable acidity (TA) decreased from 3.76 in green mature fruit to 2.79 in complete ripeness fruit. Consequently, the TSS/TA ratio (a key indicator of flavor balance) rose sharply from 1.89 in green mature fruit to 5.36 in complete ripeness, reflecting a significant shift toward sweeter and less acidic taste during ripening. Notably, vitamin C (**Figure 1C**) content was highest in green mature fruit (570.06 μg/g) and decreased slightly with ripening (542.16 μg/g in complete ripeness fruit). In summary, these results suggested significant metabolic shifts during ripening.

### 3.2 Overview of the fruit flesh metabolome analysis in *A. nanchuanensis*

The metabolomics data of *A. nanchuanensis* fruits (green mature, color-breaking, and complete ripeness) were subjected to differential analysis and identification. Principal component analysis (PCA) of metabolite profiles demonstrated clear separation among the three developmental stages, with biological replicates clustering tightly, indicating high data reliability (**Supplementary Figure 1**).

**Figure 2:**
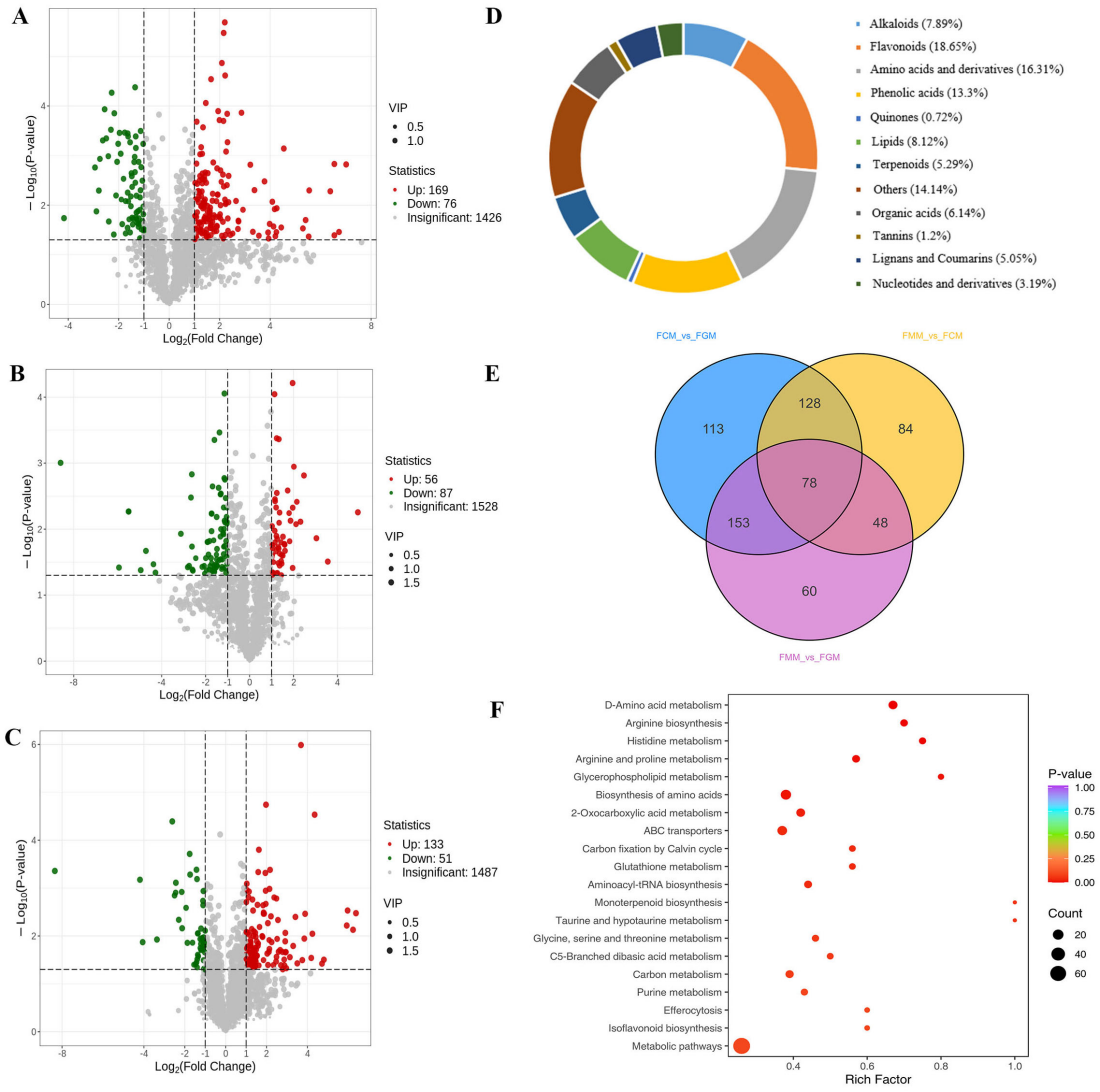
Metabolomic profiling of differentially accumulated metabolites (DAMs) during *A. nanchuanensis* fruit development. **(A)** Volcano plot of DAMs in FCM vs FGM, with red dots representing upregulated compounds. **(B)** Volcano plot of DAMs in FMM vs FCM. **(C)** Volcano plot of DAMs in FMM vs FGM. **(D)** Circular classification chart of identified metabolites in *A. nanchuanensis* fruits. **(E)** Venn diagram illustrating shared and unique DAMs among FCM vs FGM, FMM vs FCM and FMM vs FGM comparisons. **(F)** KEGG classification of 407 overlapping metabolites.

A total of 1662 compounds were successfully detected and determined in *A. nanchuanensis* fruit, categorized into 12 major classes (**Figure 2D**). The 12 class I identified compounds, including alkaloids (7.89%), amino acid derivatives (16.31%), flavonoids (18.65%), lignans and coumarin (5.05%), lipids (8.12%), nucleotide and derivatives (3.19%), organic acids (6.14%), phenolic acids (13.30%), quinones (0.72%), tannins (1.2%), terpenoids (5.29%) and other compounds (14.14%), further subdivided into 52 class II compounds. Flavonoids represented the largest proportion, while alkaloids comprised a notably high 7.89%.

**Figure 3:**
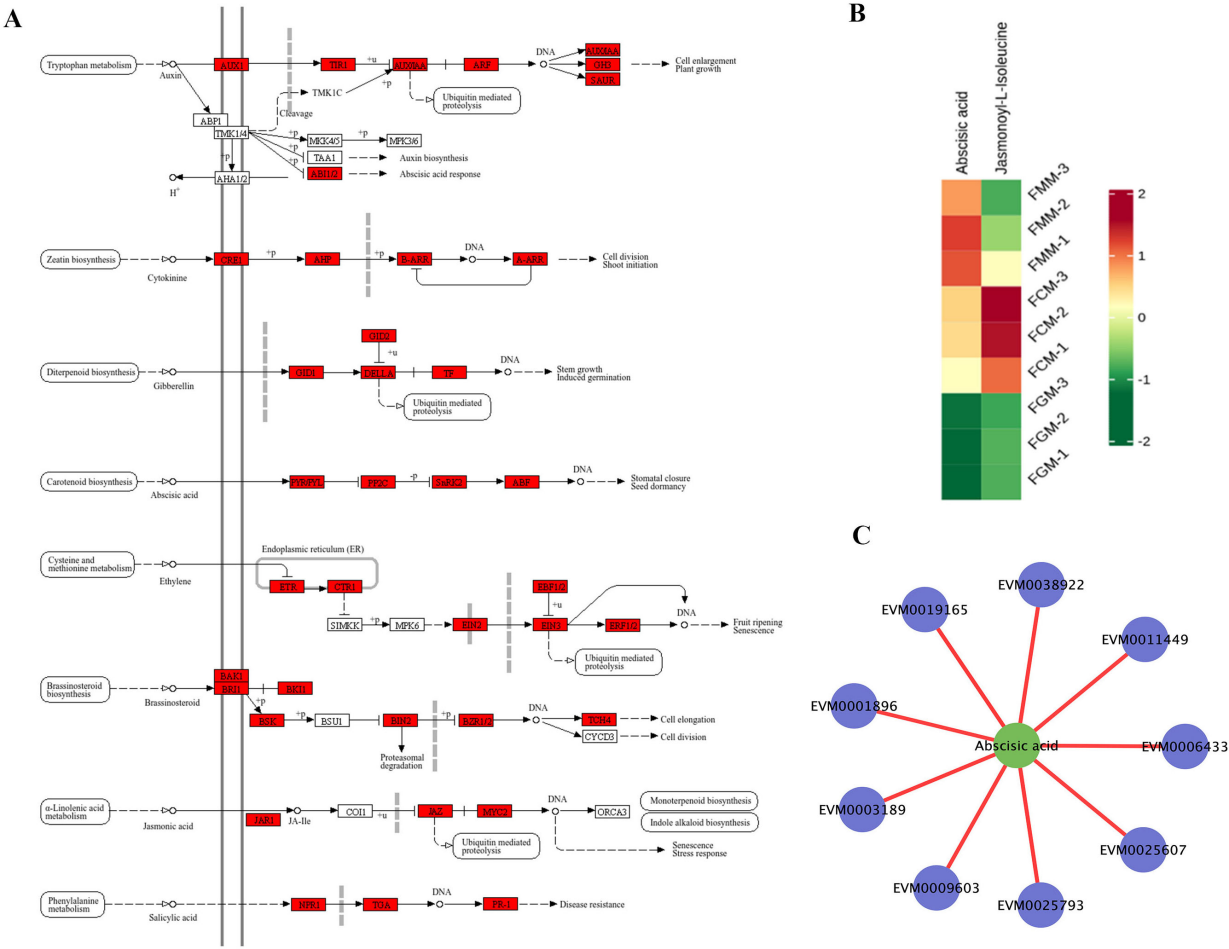
Integrated analysis of DEGs and DAMs in the plant hormone signal transduction pathway of *A. nanchuanensis* fruits. **(A)** Key DEGs were involved in the plant hormone signal transduction pathway during the ripening of *A. nanchuanensis* fruits. **(B)** Heatmap showing the relative abundance of abscisic acid (ABA) and jasmonoyl-L-isoleucine (JA-Ile). **(C)** Co-expression network analysis of ABA with selected DEGs, including transcription factors (ERF: *EVM0001896*, *EVM0003189*, *EVM0038922*; MYB: *EVM0006433*, *EVM0025793*; BES1: *EVM0009603*), an ethylene-insensitive protein (*EVM0011449*), a heat shock protein (*EVM0019165*), and a cytochrome P450 (CYP450, *EVM0025607*).

**Figure 4:**
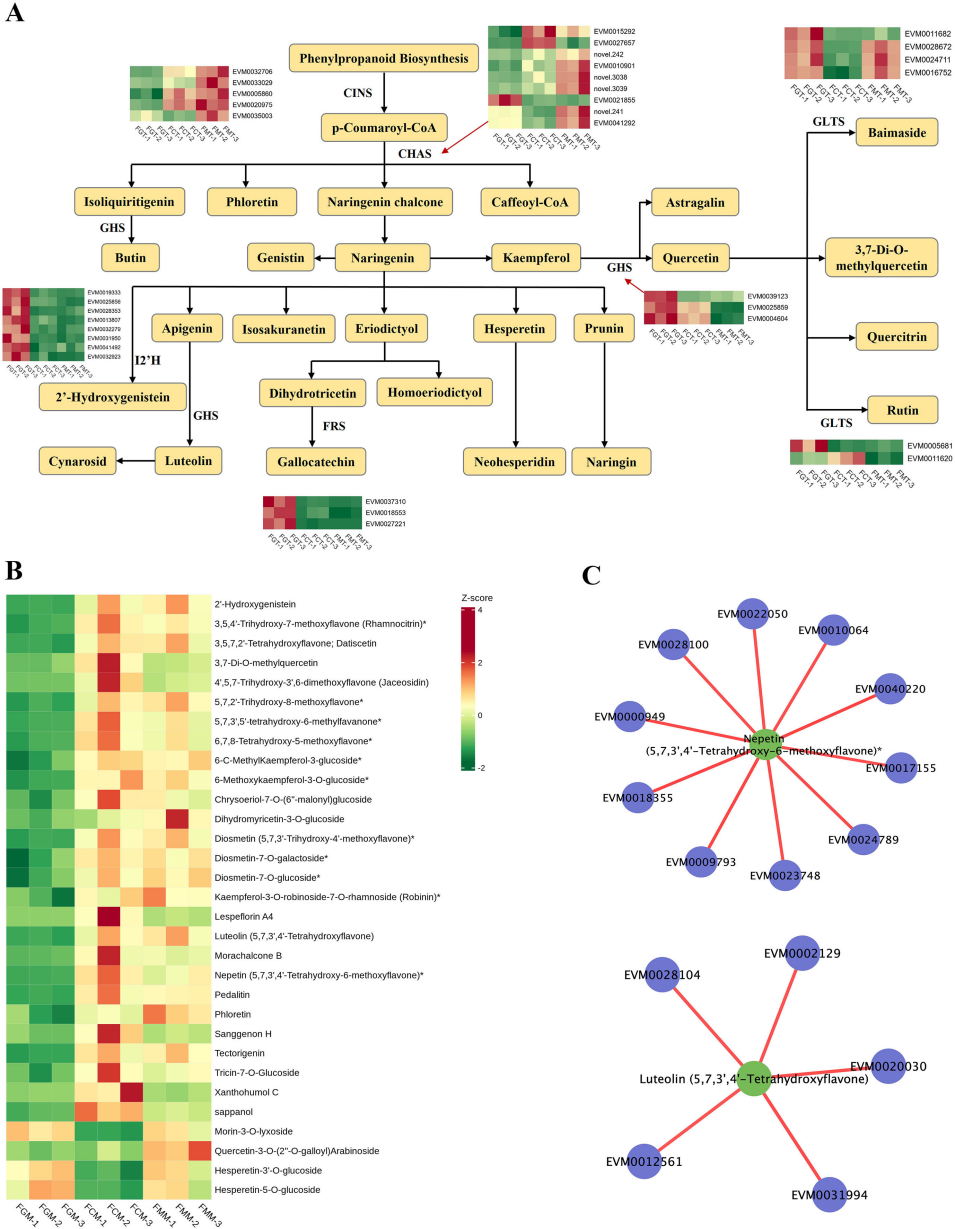
Flavonoid biosynthetic pathway related genes and differential flavonoid metabolite analysis. **(A)** Schematic representation of the flavonoid biosynthetic pathway and associated genes. **(B)** Heatmap of 31 overlapping flavonoid metabolites identified among 3 comparison groups. **(C)** Correlation network between flavonoid metabolites and select DEGs, including Nepetin and Luteolin. Key DEGs involved were: *EVM0018355* and *EVM0040220*, *major allergen Pru av 1*; *EVM0009793*, *F-box protein SKIP23*; *EVM0000949*, *WRKY*; *EVM0024789*, *glutamate decarboxylase 4*; *EVM0023748*, *galactinol-sucrose galactosyltransferase 5*; *EVM0017155*, *wall-associated receptor kinase-like 1*; *EVM0010064*, *phosphatase 2C*; *EVM0028100*, *UDP-glycosyltransferase*; *EVM0022050*, *TIFY 9*; *EVM0002129*, *receptor-like protein kinase Theseus 1*; *EVM0012561*, *1-deoxyxylulose-5-phosphate synthase DXS*; *EVM0028104*, *NB-ARC domain, LRR domain containing protein*; *EVM0031994*, *histone acetyltransferase HAC1*; *EVM0020030*, *TIR-NBS-LRR-like protein TNL*.

**Figure 5:**
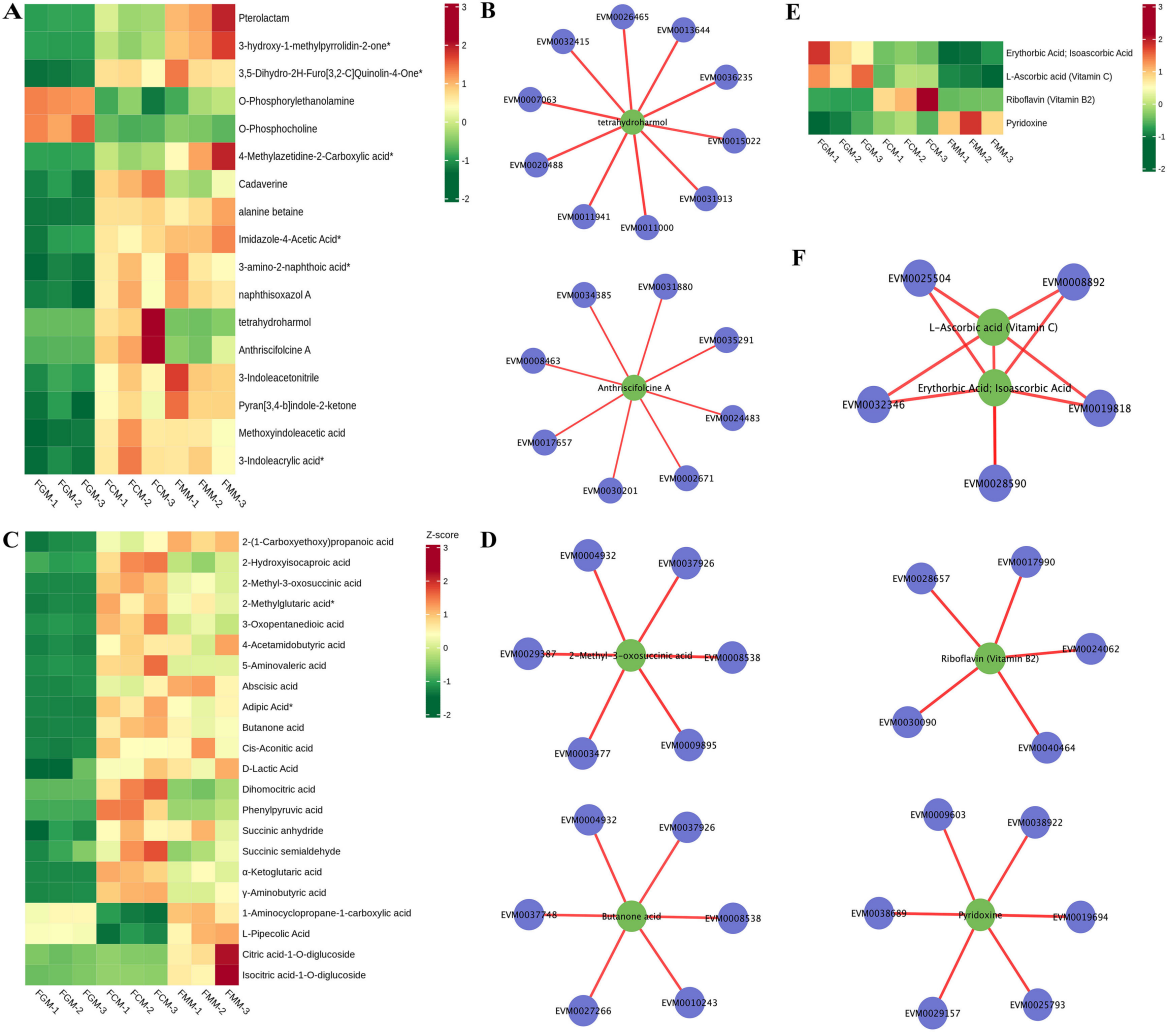
Heat map of DAMs and metabolite-DEG correlation networks. **(A)** Heatmap of 17 overlapping alkaloids identified among 3 comparison groups. **(B)** Correlation network between alkaloid metabolites and select DEGs, including tetrahydroharmol and Anthriscifolcine A. **(C)** Heat map of 22 overlapping differential accumulated organic acid, identified among 3 comparison groups. **(D)** Correlation network between organic acid metabolites and associated DEGs, including 2-Methyl-3-oxosuccinic acid, and Butanone acid. **(E)** Heat map of differential accumulated vitamin metabolites. **(F)** Correlation network between vitamin metabolites and key DEGs, including Vitamin C, Vitamin B2, Pyridoxine, and Isoascorbic Acid. Key DEGs involved in networks **(B, D, F)**: *EVM0009895*, *bHLH*; *EVM0037926*, *abscisic acid insensitive 1 protein*; *EVM0004932*, *EVM0010243*, *heat stress transcription factor, HSF*; *EVM0003477*, *EVM0027266*, *E3 ubiquitin-protein ligase*; *EVM0029387*, *DELLA protein GAI **(A)***; *EVM0037748*, *cytochrome P450*; *EVM0008538*, *E2F*; *EVM0011941*, *ABA receptor PYL4*; *EVM0015022*, *EVM0020488*, *bZIP*; *EVM0036235*, *EVM0038922, ERF*; *EVM0031913*, *EVM0028590, EVM0008892*, *WRKY*; EVM0007063, *calcium-dependent protein kinase*; *EVM0011000*, *disease resistance protein RPS2*; *EVM0026465*, *gibberellin 2-oxidase 4*; *EVM0013644*, *SAUR*; *EVM0032415, EVM0032346*, *ABC transporter C family member*; *EVM0035291*, *acetyltransferase*; *EVM0034385*, *gamma-glutamylcyclotransferase*; *EVM0031880*, *calcium permeable stress-gated cation channel*, *CSC1-like*; *EVM0002671, ABC transporter G family member*; *EVM0024483*, *Early responsive to dehydration 15*, *ERD15*; *EVM0017657*, *UDP-glycosyltransferase*; *EVM0030201*, *TMV resistance protein N-like*; *EVM0008463*, *IAA21*; *EVM0019818*, *GARP-G2-like*; *EVM0025504*, *F-box protein PP2-B15*; *EVM0019694*, *heat shock 70 kDa protein 14*; *EVM0038689*, *glutathione hydrolase 3*; *EVM0025793*, *EVM0029157*, *MYB*; *EVM0009603*, *BES1*; *EVM0040464*, *E2F*; *EVM0030090*, *B3*; *EVM0028657, ABI1*; *EVM0017990*, *Sucrose phosphate synthase*; *EVM0024062*, *ABC transporter F family member*.

Comparative analysis identified 245 DAMs in FCM vs FGM, with 169 upregulated and 76 downregulated compounds (**Figure 2A**), and 143 DAMs in FMM vs FCM (56 upregulated, 87 downregulated; **Figure 2B**), and 184 DAMs in FMM vs FGM (133 upregulated, 51 downregulated; **Figure 2C**). In this comparative analysis of the three groups, the top 10 upregulated metabolites (**Supplementary Figures 2A–C**) include organic acids, alkaloids, saccharides, amino acids and derivatives, and flavonoids. The 2-methyl-3-oxosuccinic acid, S-allyl-L-cysteine, α-ketoglutaric acid, butanone acid, and 3-oxopentanedioic acid were consistently upregulated in both FCM vs FGM and FMM vs FGM comparisons, suggesting their potential role in flavor development during *A. nanchuanensis* fruit ripening. The venn diagram illustrated overlapping and unique DAMs among FCM vs FGM, FMM vs FCM and FMM vs FGM comparisons (**Figure 2E**). KEGG classification (**Figure 2F**; **Supplementary Figure 2D**) showed 407 overlapping metabolites enriched in the metabolism pathways of saccharides (starch/sucrose metabolism, galactose metabolism, fructose and mannose metabolism), amino acids and derivatives (Histidine metabolism, Arginine biosynthesis), organic acids (butanoate metabolism, 2-Oxocarboxylic acid metabolism), alkaloids (tropane/piperidine/pyridine alkaloid biosynthesis, indole alkaloid biosynthesis), and flavonoids (phenylalanine metabolism, isoflavonoid biosynthesis, flavone and flavonol biosynthesis, flavonoid biosynthesis). Among them, saccharides (D-Lactose*, Laminaran, D-Galacturonic acid*, D-Glucoronic acid*, D-Cellobiose), flavonoids (Luteolin, Nepetin, Xanthohumol C, Morachalcone B, Sanggenon H), alkaloids (tetrahydroharmol, Anthriscifolcine A, Pterolactam) and organic acids (2-methyl-3-oxosuccinic acid, α-ketoglutaric acid, butanone acid) exhibited significantly upregulated levels in complete ripeness fruit. The dynamics of the 407 shared DAMs (**Supplementary Figures 4**, **5A** and **6**; **Figures 3B**, **4B**, **5A, C, E**) could play regulatory roles in fruit ripening, which might affect the flavor and nutritional quality of the complete ripeness fruit.

### 3.3 Transcriptome analysis of *A. nanchuanensis* fruit at three developmental stages

Based on the metabolic changes identified through widely-targeted metabolomics, transcriptomic analysis was further performed to elucidate the molecular mechanisms regulating these metabolic alterations. PCA results from the transcriptome dataset confirmed high data reliability (**Supplementary Figure 1**).

**Figure 6:**
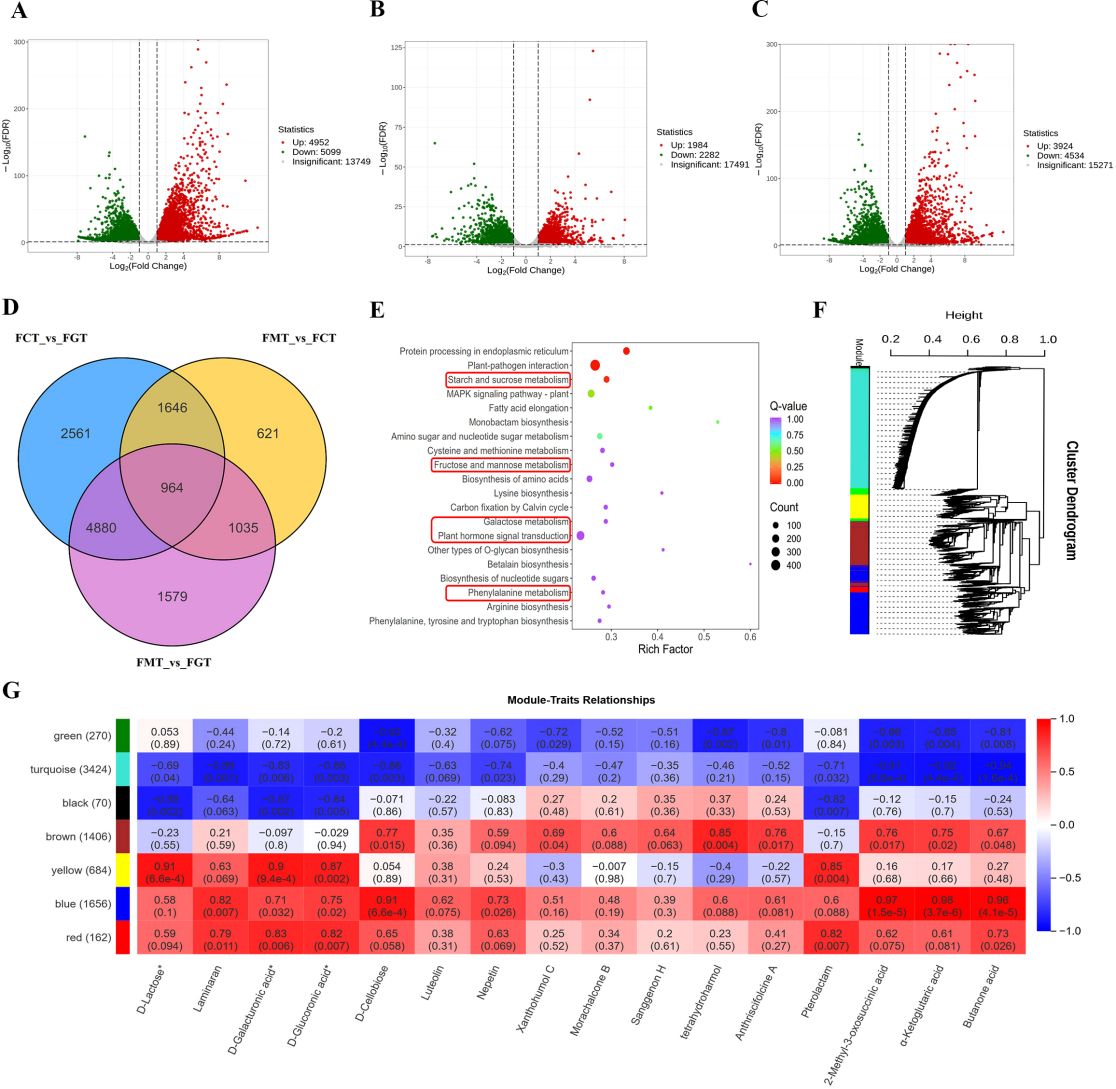
Transcriptome profiling of *A. nanchuanensis* fruit during development. **(A)** Volcano plot displaying DEGs between FCT and FGT stages. **(B)** Volcano plot displaying DEGs in FMT vs FCT. **(C)** Volcano plot displaying DEGs in FMT vs FGT. **(D)** Venn diagram illustrating shared and unique DEGs among FCT vs FGT, FMT vs FCT and FMT vs FGT comparisons. **(E)** KEGG pathway enrichment analysis of 8525 overlapping DEGs. **(F)** WGCNA module hierarchical clustering dendrogram. **(G)** Module-metabolite correlation heatmap. Deeper red and blue colors represent stronger positive and negative correlations, respectively, with lighter colors indicating weaker associations.

Comparative transcriptome analysis revealed substantial changes in gene expression patterns during fruit development. In the FCT vs FGT comparison, 23,800 expressed genes were identified, including 4,952 upregulated and 5,099 downregulated genes (**Figure 6A**). The 21,757 DEGs were identified in FMT vs FCT comparison analysis, including 1,984 upregulated and 2,282 downregulated genes (**Figure 6B**). And the FMT vs FGT comparison analysis identified 23,729 DEGs (3,924 upregulated and 4,534 downregulated; **Figure 6C**). The venn diagram of three comparison groups revealed 8525 overlapping DEGs (**Figure 6D**), which might regulate changes in metabolic substances during fruit ripening. These DEGs enriched in the pathways of starch and sucrose metabolism, fructose and mannose metabolism, biosynthesis of amino acids, galactose metabolism, plant hormone signal transduction and phenylalanine metabolism (**Figure 6E**). WGCNA analysis revealed that these 8,525 DEGs were clustered into 7 color modules (**Figure 6F**). The **Figure 6G** displayed the correlation between some significantly upregulated DAMs and color modules during fruit ripening. Saccharides (D-lactose, laminaran, D-galacturonic acid, and D-glucuronic acid*) and alkaloids (pterolactam) showed high correlations with genes in the yellow, blue, and red modules. Sugars (D-cellobiose), flavonoids (nepetin), and organic acids (2-methyl-3-oxosuccinic acid, α-ketoglutaric acid, butanone acid) exhibited strong correlations with genes in the brown, blue, and red modules. Flavonoids (xanthohumol C, morachalcone B, and sanggenon H) were highly correlated with genes in the brown module.

### 3.4 Integrative analysis of related genes and metabolites in plant hormone signal transduction pathway of *A. nanchuanensis* fruits

Among the 8,525 overlapping DEGs in **Figure 6D**, 264 DEGs were enriched in the plant hormone signal transduction pathway. The relevant genes were extracted to construct the metabolic pathways diagrams (**Figure 3A**), including auxin, cytokinin, gibberellin, abscisic acid (ABA), ethylene, brassinosteroid, jasmonic acid, and salicylic acid metabolic regulatory pathways. 138 DEGs showed significant upregulation in FCT or FMT, compared to those in FGT (**Supplementary Figure 3**).

Some genes *EVM0033560* (*AUX/IAA*), *EVM0032570* (*GH3*), *EVM0010223* and *EVM0005837* (*SAUR*), *EVM0003538* (*B-ARR*), *EVM0017295* (*GID1*), *EVM0031752* and *EVM0009222* (*DELLA*), *EVM0000157*, *EVM0014573* and *EVM0032694* (TF: *bHLH*), *EVM0031545* (*PYR/PYL*), *EVM0036043*, *EVM0011372*, *EVM0020943*, *EVM0026201* and *EVM0005070* (*PP2C*), *EVM0019444* (*SnRK2*), *EVM0007397* (*CTR1*), *EVM0011449* (*EIN2*), *EVM0026712* (*ERF1/2*), *EVM0040044* (*BAK1*), *EVM0026097* (*BRI1*), *EVM0021130* (*JAZ*), *EVM0038878* (*MYC2*), *EVM0009084* (*PR-1*), exhibited a gradually upward trend from FGT to FMT. These genes might regulate changes in plant hormone content.

The metabolomics analysis results showed that ABA levels gradually increased from FGM to FMM, whereas Jasmonoyl- L-Isoleucine was significantly upregulated only in FCM. Correlation network analysis (**Figure 3C**) between ABA and certain genes in the transcriptome revealed that ABA was positively correlated with *EVM0001896* (*ethylene-responsive transcription factor*, *ERF*), EVM0003189 (*ERF*), *EVM0006433* (*MYB*), *EVM0009603* (*brassinosteroid resistant BES1*), *EVM0011449* (*ethylene-insensitive protein*), *EVM0019165* (*heat shock protein*), *EVM0025607* (*cytochrome P450, CYP450*), *EVM0025793* (*MYB*) and *EVM0038922* (*ERF*). These results suggested that the plant hormone signal transduction pathway might play a crucial role in the ripening of *A. nanchuanensis* fruits.

### 3.5 Analysis of the regulatory pathways of saccharides metabolites

In this study, 84 saccharides metabolites were detected in *A. nanchuanensis* fruits. There were 14 saccharides compounds in the 407 overlapping metabolites of 3 comparative groups. They were all significantly upregulated in FMM, compared to those in FGM (**Supplementary Figure 4**). This indicated the accumulation of saccharides in complete ripeness fruits, which may influence their flavor. 3-Phospho-D-glyceric acid and 1-(sn-Glycero-3-phospho)-1D-myo-inositol showed change from FGM to FMM, with a decrease followed by an increase. The D-Erythrose-4-phosphate, D-Fructose-1,6-biphosphate, Ribulose-5-phosphate, Laminaran, Stachyose, D-Pinitol*, D-Galacturonic acid*, D-Glucoronic acid*, D-Lactose*, Trehalose 6-phosphate, showed gradual increase from FGM to FMM, with significantly highest levels in FMM. D-Cellobiose showed peak accumulation in FCM.

**Figure 7:**
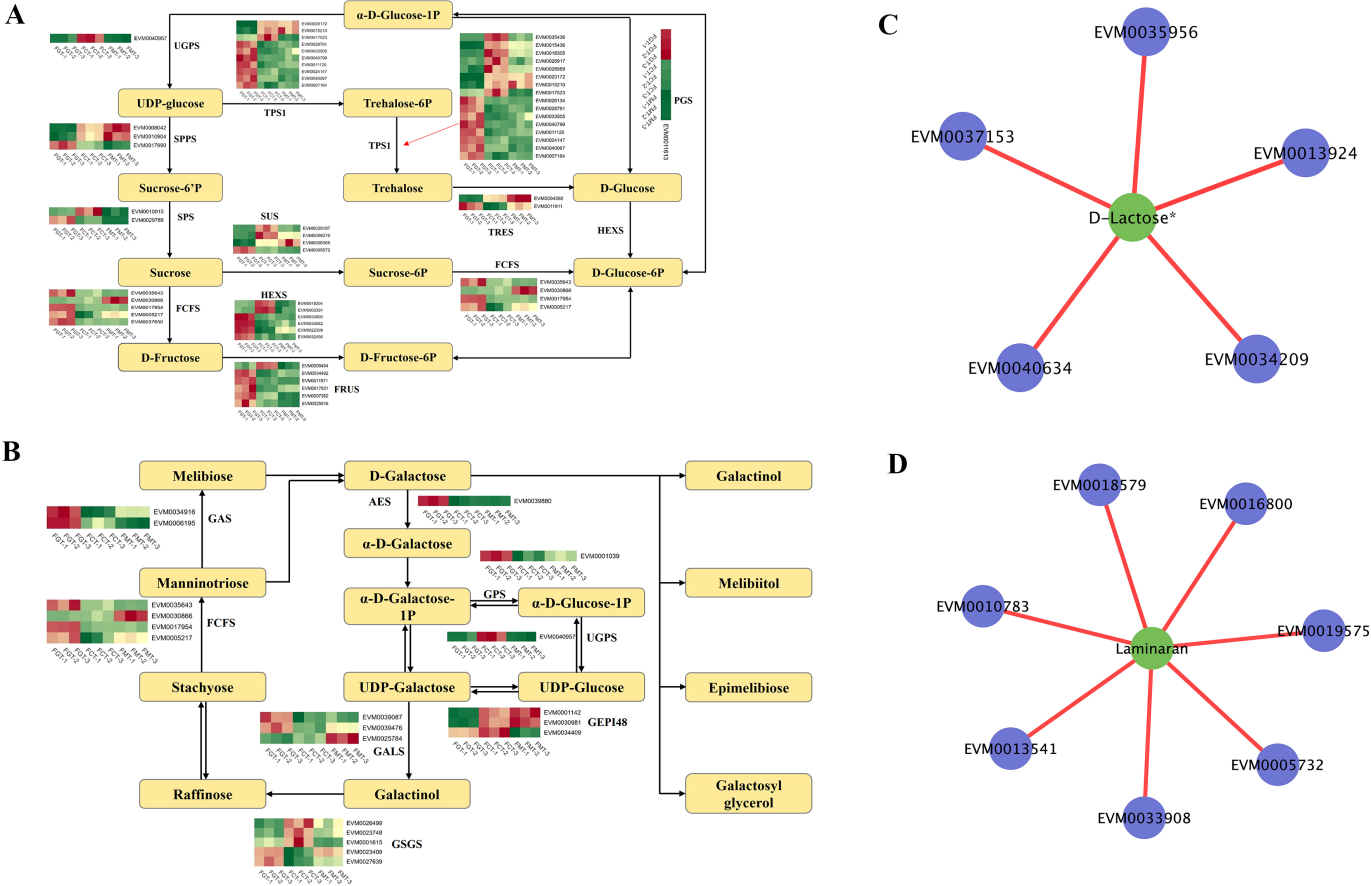
Expression heat map and saccharides metabolic biosynthesis pathways in *A. nanchuanensis* fruits. **(A, B)** Overview of saccharide metabolic biosynthesis pathways, displaying gene expression profiles across different fruit developmental stages. The heatmap was generated using Log_2_-based FPKM fold values, with red indicating high expression and green indicating low expression. **(C, D)** Correlation network between saccharide metabolites and select DEGs, including Laminaran and D-Lactose*. The associated DEGs were: *EVM0018579*, *E3 ubiquitin ligase*; *EVM0016800*, *gibberellin 2-beta-dioxygenase 2*; *EVM0005732*, *F-box/LRR-repeat protein 4*; *EVM0010783*, *toll-like receptor 3*; *EVM0013541*, *multiprotein-bridging factor 1b*; *EVM0033908*, *CAX-interacting protein 4*; *EVM0019575*, *ubiquitin-conjugating enzyme E2*; *EVM0013924*, *SIEVE ELEMENT OCCLUSION B*; *EVM0035956*, *mitochondrial outer membrane import complex protein METAXIN*; *EVM0040634*, *histidine kinase 2*; *EVM0034209, peptidyl-prolyl cis-trans isomerase; EVM0037153, ABC transporter C family member*.

The regulation of starch and sucrose metabolism, as well as galactose metabolism, was further explored (**Figures 7A, B**). In the D-Glucose-6P biosynthesis pathway, 21 single genes were identified, encoding seven putative enzymes, including Sucrose phosphate synthase (SPPS), Hexokinase (HEXS), sucrose-phosphatase 2 (SPS), sucrose synthase 2 (SUS), phosphoglucomutase (PGS), and UDP-glucose pyrophosphorylase (UGPS). In the pathway converting sucrose to D-Fructose-6P, 17 single genes encoded three putative enzymes: beta-fructofuranosidase (FCFS), HEXS, and fructokinase (FRUS). UDP-glucose was transformed into Trehalose-6P and Trehalose through the action of TPS1 enzymes encoded by 16 genes. Additionally, D-Galactose was converted to α-D-Glucose-1P via aldose 1-epimerase (AES) and Galactose-1-phosphate uridylyltransferase (GPS), while UDP-Galactose was converted to Stachyose by galactinol synthase (GALS) and galactinol--sucrose galactosyltransferase (GSGS). The dynamic changes of these related genes in starch and sucrose metabolism and galactose metabolism pathway, might also cause the changes of these 14 saccharides compounds.

Integrated transcriptome-metabolome correlation analysis revealed several notable associations (**Figures 7C, D**). Laminaran exhibited positive correlations with *EVM0018579* (*E3 ubiquitin ligase*), *EVM0016800* (*gibberellin 2-beta-dioxygenase 2*), *EVM0005732* (*F-box/LRR-repeat protein 4*), *EVM0010783* (*toll-like receptor 3*), *EVM0013541* (*multiprotein-bridging factor 1*), *EVM0033908* (*CAX-interacting protein 4*) and *EVM0019575* (*ubiquitin-conjugating enzyme E2*). D-Lactose* was positively correlated with *EVM0013924* (*SIEVE ELEMENT OCCLUSION B*), *EVM0035956* (*mitochondrial outer membrane import complex protein*), *EVM0040634* (*histidine kinase 2*), *EVM0034209* (*peptidyl-prolyl cis-trans isomerase*) and *EVM0037153* (*ABC transporter C family member*). In addition, stachyose (**Supplementary Figure 5**) showed positive associations with *EVM0034564* (*auxin-responsive protein*, *SAUR71*), *EVM0031968* (*MYB*), and *EVM0020005* (*ERF*). D-Pinitol* (**Supplementary Figure 5**) correlated positively with *EVM0015262* (*CYP450*), *EVM0017439* (*heat shock protein*), and *EVM0040255* (*bZIP*). This multi-omics integration highlighted the complex regulatory networks governing saccharide metabolism during fruit ripening in *A. nanchuanensis*.

### 3.6 Analysis of the regulatory pathways of flavonoids metabolites

In *A. nanchuanensis* fruits, flavonoids were the main metabolites, with a total of 310 substances. There were 62 differential accumulated flavonoids in the 407 overlapping metabolites of 3 comparative groups. Among them, 31 flavonoids showed significantly upregulated in FMM, compared to those in FGM (**Figure 4B**). The Morin-3-O-lyxoside, Hesperetin-3’-O-glucoside and Hesperetin-5-O-glucoside revealed a trend of first decreasing and then increasing from FGM to FMM. The Phloretin, Quercetin-3-O-(2’’-O-galloyl) Arabinoside, Robinin, and Dihydromyricetin-3-O-glucoside displayed highest relative levels in FMM, compared to those in FGM and FCM. Other flavonoids, such as Morachalcone B, Lespeflorin A4, Sanggenon H and Xanthohumol C, peaked at FCM. But complete ripeness fruits also contain abundant amounts of flavonoids.

Phenylalanine serves as the precursor for flavonoid biosynthesis, with key enzymes such as PAL, C4H, 4CL, and CHS driving its conversion into various flavonoid metabolites. In the analysis of the metabolites in *A. nanchuanensis* fruits, there were 13 flavonoids compounds, mapped to the phenylpropanoid-flavonoid pathways (**Figure 4A**), including Luteolin, Luteolin-7-O-glucoside (Cynaroside)*, Astragalin, Naringenin-7-O-Neohesperidoside (Naringin)*, 3,7-Di-O-methylquercetin, Rutin, Quercetin-3-O-sophoroside (Baimaside)*, Phloretin, Quercetin-3-O-rhamnoside (Quercitrin), Hesperetin-7-O-neohesperidoside (Neohesperidin)*, (+)-Gallocatechin, 2’-Hydroxygenistein, Genistein-7-O-Glucoside (Genistin). In addition, 6 enzymes were identified to be involved in the molecules modulating of the phenylpropanoid-flavonoid pathways, including trans-cinnamate 4-monooxygenase (CINS), Isoflavone 2’-hydroxylase (I2’H), Flavonol reductase/cinnamoyl-CoA reductase (FRS), chalcone synthase (CHS), and UDP-glucuronosyl and UDP-glucosyl transferase (GLTS), geraniol 8-hydroxylase (GHS), with their activity governed by 34 identified genes. The transcription levels of these genes might regulate 31 flavonoids dynamic changes. Notably, the transcription levels of three *GHS* genes exhibited downregulated from FGT to FMT, suggesting negative regulatory roles in the conversion of apigenin to luteolin.

In addition, the combined analysis of transcriptome and metabolome revealed Nepetin was positively correlated with some genes (**Figure 4C**), such as *EVM0018355*, *EVM0040220* (*major allergen Pru av 1*), *EVM0000949* (*WRKY*), *EVM0017155* (*wall-associated receptor kinase-like 1*), *EVM0010064* (*phosphatase 2C*), etc. Luteolin showed positive associations with *EVM0031994* (*histone acetyltransferase HAC1*), *EVM0020030* (*TIR-NBS-LRR-like protein TNL*), *EVM0028104* (*NB-ARC domain, LRR domain containing protein*), *EVM0002129* (*receptor-like protein kinase THESEUS 1*) and *EVM0012561* (*1-deoxyxylulose-5-phosphate synthase DXS*). Xanthohumol C (**Supplementary Figure 5**) was positively correlated with *EVM0030090* (*SAUR18*), *EVM0008463* (*IAA21*) and *EVM0019052* (*WRKY*). Both Morachalcone B and Sanggenon H (**Supplementary Figure 5**) displayed positive relationships with *EVM0031235* (*MYB*).

### 3.7 Heat map analysis of alkaloids metabolites accumulation

Alkaloids are nitrogen-containing bioactive compounds with significant pharmacological importance. A total of 131 alkaloid metabolites were identified in *A. nanchuanensis* fruit, with 31 overlapping among 3 comparison groups. Among them, 18 alkaloid compounds showed significant upregulated accumulation in FMM, compared to those in FGM (**Figure 5A**). The Fagomine, Guanidinoacetate, Dihydroisopelletierine showed lowest relative levels in FCM, compared to those in FGM and FMM. Tetrahydroharmol exhibited the most pronounced content change, with its content in FCM being 197-fold higher than in FGM, followed by Anthriscifolcine A (14.4-fold increase in FCM). Additionally, 3-hydroxy-1-methylpyrrolidin-2-one*, 4-Methylazetidine-2-Carboxylic acid* and Pterolactam were significantly enriched in FMM.

The combined analysis of transcriptome and metabolome found that tetrahydroharmol exhibited strong positive correlations (**Figure 5B**) with *EVM0011941* (*ABA receptor PYL4*), *EVM0015022* (*bZIP*), *EVM0036235* (*ERF*), *EVM0031913* (*WRKY*), *EVM0011000* (*disease resistance protein RPS2*), *EVM0013644* (*SAUR*), etc. Anthriscifolcine A showed positive associations with *EVM0035291* (*acetyltransferase*), *EVM0031880* (*calcium permeable stress-gated cation channel*, *CSC1-like*), *EVM0002671* (*ABC transporter G family member*), *EVM0024483* (*Early responsive to dehydration 15*, *ERD15*), *EVM0030201* (*TMV resistance protein N-like*), *EVM0008463* (*IAA21*), etc. Pterolactam (**Supplementary Figure 5**) shared correlations with *EVM0008463* (*IAA21*) and *EVM0030201* (*TMV resistance protein N-like*). These findings suggested intricate regulatory networks governing alkaloid biosynthesis and stress adaptation. However, the complete metabolic network map requires further elucidation.

### 3.8 Differential accumulation for organic acid, vitamin and amino acids and derivatives

A total of 102 organic acid metabolites were identified in *A. nanchuanensis* fruit, with 31 overlapping accumulation during 3 comparison groups. The heat map of organic acid relative levels (**Figure 5C**) displayed 22 significant accumulation compounds in FMM. The Citric acid-1-O-diglucoside, Isocitric acid-1-O-diglucoside, 2-(1-Carboxyethoxy) propanoic acid, Abscisic acid, 1-Aminocyclopropane-1-carboxylic acid, L-Pipecolic Acid were significant upregulation in FMM. Notably, 2-Methyl-3-oxosuccinic acid demonstrated the most dramatic upregulation, with its relative levels in FCM and FMM reaching 129.1-fold and 82.7-fold higher, respectively, compared to FGM levels. The changes in these organic acids might affect the flavor of ripe fruits.

In the integrated transcriptomic and metabolomic analysis (**Figure 5D**), 2-Methyl-3-oxosuccinic acid showed positive correlations with *EVM0009895* (*bHLH*), *EVM0008538* (*E2F*), *EVM0004932* (*HSF*), *EVM0037926* (*ABI1*), *EVM0003477* (*E3 ubiquitin-protein ligase*) and *EVM0029387*, *EVM0003285* [*DELLA protein GAI (A)*]. Similarly, α-Ketoglutaric acid, Butanone acid, and 3-Oxopentanedioic acid were also positively correlated with *EVM0008538* (*E2F*) and *EVM0004932* (*HSF*) (**Figure 5F**; **Supplementary Figure 5**). Dihomocitric acid (**Supplementary Figure 5**) exhibited positive correlations with *EVM0026465* (*gibberellin 2-oxidase 4*), *EVM0011941* (*abscisic acid receptor PYL4*), *EVM0032762* (*WRKY*), and *EVM0033998* (*MYB*).

A total of 18 vitamin metabolites were identified in *A. nanchuanensis* fruit. The dynamic changes of Vitamin C, Vitamin B2, Pyridoxine, and Erythorbic Acid; Isoascorbic Acid during fruit development were illustrated in **Figure 5E**. Erythorbic acid and vitamin C exhibited the highest accumulation levels during the FG stage. Pyridoxine peaked in the FM stage. Riboflavin (vitamin B2) followed a biphasic trend, first increasing and then decreasing, with maximal accumulation occurring in the FC stage. Notably, the developmental changes in vitamin C from FG to FM aligned with quantitative validation results (**Figure 1**).

Isoascorbic Acid and L-Ascorbic acid (Vitamin C) were positively correlated with (**Figure 5F**) *EVM0028590, EVM0008892* (*WRKY*), *EVM0019818* (*GARP-G2-like*), *EVM0032346* (*ABC transporter C family member*), *EVM0025504* (*F-box protein PP2-B15*). Pyridoxine showed positive correlations with *EVM0019694* (*heat shock 70 kDa protein 14*), *EVM0025793*, *EVM0029157* (*MYB*), *EVM0038922* (*ERF*), etc. Riboflavin (Vitamin B2) was positively associated with *EVM0040464* (*E2F*), *EVM0030090* (*B3*), *EVM0028657* (*ABI1*), *EVM0017990* (*Sucrose phosphate synthase*) and *EVM0024062* (*ABC transporter F family member*).

In *A. nanchuanensis* fruits, 271 amino acids and derivatives were identified. Among the intersection of the three differential comparison groups, 80 amino acids and derivatives were found, whose dynamic changes may affect the nutritional value of complete ripeness fruits. Of these, 20 amino acids and derivatives were significantly upregulated in FMM (**Supplementary Figure 5**). Notably, L-Proline*, 1-Amino-1-cyclobutane-carboxylic-acid*, Arg-Gly, Asp-Met-Tyr, D-Allo-Isoleucine*, His-Tyr-Glu, N-Acetyl-L-glutamic acid, N-Propionylglycine, and Pro-Asp exhibited significantly higher levels in FMM compared to FGM and FCM.

## 4 Discussion

Recent advances in bioinformatics have enabled multi-omics approaches to study fruit quality traits and metabolic networks. Transcriptomics helps identify functional genes and metabolic pathways, while metabolomics detects tissue-specific metabolite profiles using both targeted and untargeted methods (Xiao et al., 2021). Here, we combined widely-targeted metabolomics and transcriptomics to investigate key genes and metabolites during *A. nanchuanensis* fruit development, revealing molecular mechanisms underlying fruit growth and ripening.

### 4.1 Effects of saccharides, organic acids, and amino acids on the flavor of *A. nanchuanensis* fruit

During fruit development, the intricate interplay of metabolites and genes undergoes significant changes (Li et al., 2022). As assimilates are allocated to fewer fruits, sugar accumulation is enhanced, leading to improved sweetness. Fruit sweetness is primarily influenced by soluble sugars, while acidity is largely governed by organic acids. Flavor, on the other hand, depends on the composition of volatile compounds, including sugars, organic acids, and amino acids (Li et al., 2021). In ripe fruits, the total sugar content consists mainly of three components: fructose, glucose, and sucrose (Li et al., 2022). 23 sugars were screened from the pulp of the Korla fragrant pear fruits, and D- lactose was significantly different in fruits of different appearances (Jiang et al., 2023). Most sugars accumulated gradually during hawthorn fruit growth, with their accumulation rate significantly accelerating during ripening (Wang et al., 2023). To date, no studies have explored the influence of secondary metabolites on *A. nanchuanensis* fruit flavor. The HPLC-MS/MS analysis showed that, a total of 84 saccharides were identified. 14 saccharides compounds in the overlapping metabolites of 3 comparative groups (FMM vs FGM, FCM vs FGM and FMM vs FCM) were significant upregulation in FMM, which were likely key contributors to flavor. The sucrose synthase (SUS), sucrose phosphate synthase (SPPS), UDP-glucose pyrophosphorylase (UGPS), also regulated D-glucose-6P biosynthesis during *A. nanchuanensis* fruit development. Previous study has shown that the sugar content of the watermelon fruit is mainly determined by three enzyme families, SUS, sucrose-phosphatase 2 (SPS) and insoluble acid convertase (IAI) (Yuan et al., 2022). SUS can catalyze both sucrose synthesis and sucrose catabolism, but mainly convert UDP-glucose into sucrose (Schmölzer et al., 2016). In tomato fruit, SUS contributes to the accumulation of glucose and fructose (Li et al., 2021). These findings indicated that variations in saccharides metabolism-related genes might modulate sugar accumulation in *A. nanchuanensis* fruit, thus affecting their flavor.

Organic acids in fruits improve flavor, color, and aroma. Different fruits contain varying types and levels of organic acids, creating unique tastes (Bouhlali et al., 2020). In *A. nanchuanensis* fruits, 102 organic acids were detected. 22 overlapping accumulation during 3 comparison groups were increased significantly in complete ripeness fruits. Although some organic acids, including 2-Methyl-3-oxosuccinic acid, Butanone acid, α-Ketoglutaric acid, and 3-Oxopentanedioic acid, showed highest levels in FCM, the change of these substances also influence the flavor of complete ripeness fruits. The TSS of *A. nanchuanensis* complete ripeness fruit was 14.95%, and TA was 2.79%. Variations in sugar and organic acid metabolites alter TSS and TA in *A. nanchuanensis* fruits, ultimately affecting flavor. The metabolomic analysis revealed distinct metabolic profiles underlying the flavor differences among three apricot cultivars: ‘Shushanggan’ accumulated higher levels of sucrose, glucose, fructose, and sorbitol but lower citric acid and titratable acid, resulting in superior flavor; ‘Sungold’ accumulated more sucrose alongside less citric acid and starch, contributing to its distinct flavor; and ‘F43’ accumulated higher titratable acid, citric acid, and starch, leading to a lower degree of sweetness (Gou et al., 2023).

The composition and abundance of amino acids are crucial indicators of nutritional quality and flavor (Choi et al., 2012). 271 amino acids and derivatives were identified in *A. nanchuanensis* fruits. Of these, L-Proline*, 1-Amino-1-cyclobutane-carboxylic-acid*, Arg-Gly, Asp-Met-Tyr, D-Allo-Isoleucine*, His-Tyr-Glu, N-Acetyl-L-glutamic acid, N-Propionylglycine, and Pro-Asp exhibited significantly higher levels in FMM compared to FGM and FCM. These findings indicate that variations in saccharides, organic acids, and amino acids likely contribute to differences in fruit flavor and nutritional quality.

### 4.2 Flavonoids biosynthesis in *A. nanchuanensis* fruit

Flavonoids, the most abundant secondary metabolites, include flavonols, flavanones, and isoflavones, and are widely present in fruits and vegetables (Zhang et al., 2017). They influence plant color, development, nutritional quality, and environmental adaptation (Sagawa et al., 2016), producing hues ranging from yellow to red and pale yellow to orange (Ni et al., 2020). Their accumulation affects fruit pigmentation, such as mango color and apple peel composition (Peng et al., 2022), and citrus peels and tomatoes are rich in flavanones (Wang et al., 2021). In an integrated transcriptomic and metabolomic analysis conducted on three jackfruits (*Artocarpus heterophyllus*) cultivars—light-yellow “THA,” yellow “GTM,” and orange “YNH”—a total of twenty-five differentially accumulated flavonoids were identified. Among these, naringenin chalcone, eriodictyol, and taxifolin were found to be the key flavonoids responsible for the light-yellow coloration of THA pulp. Phlorizin was associated with the yellow hue of GTM pulp, while apigenin and luteolin were identified as the primary pigments contributing to the orange tone of YNH pulp (Liang et al., 2026). Untargeted metabolomic profiling of roots, stems, and leaves from *Areca catechu* (betel nut) identified 331 metabolites, with flavonoids representing the largest class (107 compounds) (Lai et al., 2023). In *A. nanchuanensis* fruits, flavonoids were the predominant metabolites, with a total of 310 types. Given the yellow peel and pulp at maturity, flavonoids likely drive fruit yellowing, though further validation is needed. Additionally, naringin and neohesperidin contribute to citrus bitterness (Wang et al., 2016). In *A. nanchuanensis* fruits, phloretin rose continuously from FGM to FMM, while nepetin surged up to 11.3-fold in FMM vs FGM. And 31 differential accumulated flavonoids in the intersection of 3 comparative groups showed significantly upregulated in FMM, which may also influence *A. nanchuanensis* flavor. But many flavonoids exhibited highest levels in FCM, which helped with the extraction of functional components.

Transcriptomic analysis in this study revealed some DEGs in phenylpropanoid-flavonoid pathways, including six key enzymes: trans-cinnamate 4-monooxygenase (CINS), Isoflavone 2’-hydroxylase (I2’H), Flavonol reductase/cinnamoyl-CoA reductase (FRS), chalcone synthase (CHS), UDP-glucuronosyl and UDP-glucosyl transferase (GLTS), and geraniol 8-hydroxylase (GHS). Notably, CHS, the first enzyme in flavonoid biosynthesis, showed upregulated expression in FMT vs FGT. CHS catalyzes p-Coumaroyl-CoA conversion to naringenin chalcone, isoliquiritigenin, phloretin, and caffeoyl-CoA, which further isomerizes to naringenin (Chaudhary et al., 2016). In jackfruit, differentially expressed genes CHS, were also detected through combined analysis of transcriptomics and metabolomics (Liang et al., 2026).

Flavonoids biosynthesis are regulated by key transcription factors (TFs), including MYB, bHLH, and WRKY, which form MBW complexes to modulate phenylpropanoid and proanthocyanin production (Nemie-Feyissa et al., 2015; Fernandez-Moreno et al., 2016). Integrated analysis of the metabolome and transcriptome in betel nut suggested that the transcription factors AcMYB5 and AcMYB194 may play a regulatory role in flavonoid biosynthesis (Lai et al., 2023). SiMYB12 in pink tomato influences flavonoid metabolism, and AtWRKY23 in Arabidopsis enhances flavonoid biosynthesis by upregulating flavonoid biosynthesis pathway enzymes genes (Grunewald et al., 2012). In *Actinidia arguta*, AaMYB and AabHLH have also been reported to regulate flavonoid biosynthesis (Ye et al., 2023). The abnormal expression of bHLH3 in mulberry fruits disrupts the flavonoid metabolic balance, altering the levels and ratios of anthocyanins, flavones, and flavonols, which contributes to variations in fruit color (Li et al., 2020). In this study, multiple differentially expressed MYB (EVM0031235), WRKY (EVM0000949 and EVM0019052), and bHLH (EVM0009895) TFs were identified, potentially influencing flavone/isoflavone biosynthesis during fruit ripening. However, further experimental validation is needed to confirm their regulatory roles.

### 4.3 Alkaloids and vitamin biosynthesis in *A. nanchuanensis* fruit

Alkaloids represent a structurally diverse class of bioactive compounds with significant pharmaceutical applications, particularly as antibacterial agents (Yan et al., 2021). Metabolomic analysis revealed that alkaloids accounted for 10.28% of the total metabolites in jackfruit, including plumerane, pyridine alkaloids, and others (Liang et al., 2026). In *A. nanchuanensis* fruits, we identified 131 alkaloid compounds, including Plumerane, Phenolamine, Pyrrole alkaloids, Quinoline alkaloids, Pyridine alkaloids. Our analysis revealed remarkable concentration differences: Tetrahydroharmol (a Plumerane alkaloid) showed 197-fold (FCM) and 11.4-fold (FMM) increases compared to FGM. Anthriscifolcine A, a quinolizidine alkaloid, was elevated 14.5-fold (FCM) and 4.4-fold (FMM). Alkaloids, plumerane, phenolamine, pyridine alkaloids, have been reported in the mature flesh of jackfruit (Liang et al., 2026). Alkaloids have also been reported in the leaves of *Artocarpus chama* and *Artocarpus hirsutus Lam* (Hossen et al., 2025; Karthik et al., 2022). However, no specific alkaloid substances have been reported. The most frequently reported in mulberry are mulberry twig alkaloid, mainly including 1-deoxynojirimycin, fagopyrin, and 1,4-dideoxy-1,4-iminod-arabinitol. 1-Deoxynojirimycin (DNJ) is the most representative star compound (Chen et al., 2025). Anthriscifolcine A is a novel C18-diterpenoid alkaloid isolated from *Delphinium anthriscifolium* (Ranunculaceae), which has also been identified in the fruits of *A. nanchuanensis* and has not been reported in other Moraceae species to date (Song et al., 2011). Its identification in *A. nanchuanensis* therefore represents a novel finding for the family. Other significantly upregulated alkaloids in FMM included pyrrole alkaloids (Pterolactam and 3-hydroxy-1-methylpyrrolidin-2-one*), quinoline alkaloids (3,5-Dihydro-2H-Furo[3,2-C] Quinolin-4-One*), 4-Methylazetidine-2-Carboxylic acid*, plumerane (3-Indoleacetonitrile and Pyran[3,4-b] indole-2-ketone). Tetrahydroharmol exhibited strong positive correlations with EVM0015022 (bZIP), EVM0036235 (ERF), EVM0031913 (WRKY), etc. Metabolomic analysis of *Sophora alopecuroides* led to the identification of sixty-eight alkaloids, predominantly of the quinolizidine type. Furthermore, an integrated analysis of the metabolome and transcriptome revealed a positive correlation between the biosynthesis of these alkaloids and 22 transcription factors, including members of the ERF (SaERF1–SaERF5), WRKY (SaWRKY1–SaWRKY3), bHLH (SabHLH1–SabHLH3), MYB (SaMYB1–SaMYB6), and bZIP (SabZIP1–SabZIP5) families (Huang et al., 2024).

Vitamins are essential micronutrients that support normal physiological functions in humans and animals. Among these, vitamin C (L-ascorbic acid) plays crucial roles in preventing scurvy, enhancing immunity, and maintaining vascular and bone health (Rey et al., 2020). As a potent antioxidant, vitamin C serves dual functions: it acts as a key quality marker in fruits while participating in fundamental plant developmental processes including cell division and expansion (Chen et al., 2019). Jackfruit has significant nutritional value, containing vitamin C (7 mg 100 g-1 FW), along with vitamin A (540 IU per 100 g). It also provides essential B vitamins including niacin (12.7 mg per 100 g), riboflavin (130 μg per 100 g), and thiamin (39 μg per 100 g), in addition to high levels of essential minerals (Kaur et al., 2024). *A. nanchuanensis* fruit contains 18 important vitamins, such as riboflavin (B2), isoascorbic acid, vitamin C, and pyridoxine, etc. The vitamin C content of lemons is 305.75 μg/g (Sir Elkhatim et al., 2018). The vitamin C content in the fruits of the black mulberry genotype ranges from 101.23 to 162.93 μg/g, while the vitamin C content in the white mulberry ranges from 134 to 182.2 μg/g (Eyduran et al., 2015). Notably, vitamin C content of *A. nanchuanensis* complete ripeness fruit (542.16 μg/g) surpasses those of lemons, Jackfruit and mulberry. However, vitamin C content was highest in green mature fruit (570.06 μg/g) and decreased slightly with ripening. It has been observed that vitamin and mineral levels of jackfruit rise during the early phases of fruit development, but subsequently decline as the ripening process advances (Ranasinghe, 2019). These findings highlight the significant nutritional and medicinal value of *A. nanchuanensis* fruits, though further research is needed for comprehensive validation.

While our correlation and WGCNA analyses have identified several transcription factors (e.g., MYB, ERF, and WRKY) as strong candidates regulating the biosynthesis of nutritional components, we acknowledge that these findings remain predictive and require further functional validation. Future studies using approaches such as transient expression assays or virus-induced gene silencing (VIGS) or qRT-PCR assays will be necessary to confirm the specific functions of these candidate genes.

It is important to note that the fruit samples analyzed in this study were collected from 15-year-old cultivated trees. Plant metabolic profiles are known to be affected by environmental conditions. Accordingly, the absolute concentrations and even the relative dynamic changes of nutritional components such as flavonoids, alkaloids, and vitamins reported in this study may differ in fruits from natural populations of *A. nanchuanensis*, which are exposed to distinct ecological pressures. Future comparative metabolomic studies between artificially propagated individuals and wild populations would be valuable for understanding the degree of phenotypic plasticity in this critically endangered species and for guiding its conservation and utilization strategies.

## 5 Conclusion

In this study, 1,662 metabolites were identified in *A. nanchuanensis* fruits, through widely targeted metabolomic analyses. The flavonoids accounted for the highest proportion, reaching 18.65%. A total of 131 alkaloids were identified, accounting for 7.84%. In the complete ripeness fruits, flavonoids (Nepetin and Luteolin) and alkaloid (pterolactam and tetrahydroharmol) were significantly upregulated compared to those in the green mature fruits. The complete ripeness fruit was also rich in saccharides, organic acids, amino acids and vitamins. Vitamin C content was highest in green mature fruit (570.06 μg/g) and decreased slightly with ripening (542.16 μg/g in complete ripeness fruit). Integrating metabolomic and transcriptomic data, the biosynthetic pathways of flavonoids and saccharides were constructed. Some transcription factors, such as MYB, ERF, and WRKY, were potentially regulate the biosynthesis of saccharides, flavonoids, alkaloids, organic acids, and vitamins. Candidate genes associated with the synthesis of these metabolites were also uncovered. Most flavonoids reached their peak concentrations at the color-breaking stage, suggesting this may be the optimal period for their industrial extraction. Future studies should focus on the functional phenotypic characterization of metabolites such as flavonoids and alkaloids, including DPPH and ABTS assays for evaluating antioxidant capacity. These findings elucidate the nutrient composition and regulatory networks within *A. nanchuanensis* fruits, thereby paving the way for advanced studies on nutrient regulation and potential medicinal development.

## Data Availability

The datasets presented in this study can be found in online repositories. The names of the repository/repositories and accession number(s) can be found in the article/**Supplementary Material**.
