# Supplementary material for: Integrated widely-targeted metabolome and transcriptome analysis provide novel insights into the regulation of nutritional formation in Artocarpus nanchuanensis fruit
Source: Front Plant Sci. 2026 Apr 13;17:1800089. doi: 10.3389/fpls.2026.1800089 (PMC13111455; doi:10.3389/fpls.2026.1800089)
Supplement: Supplementary file 1 [file DataSheet1.docx]

Supplementary Material


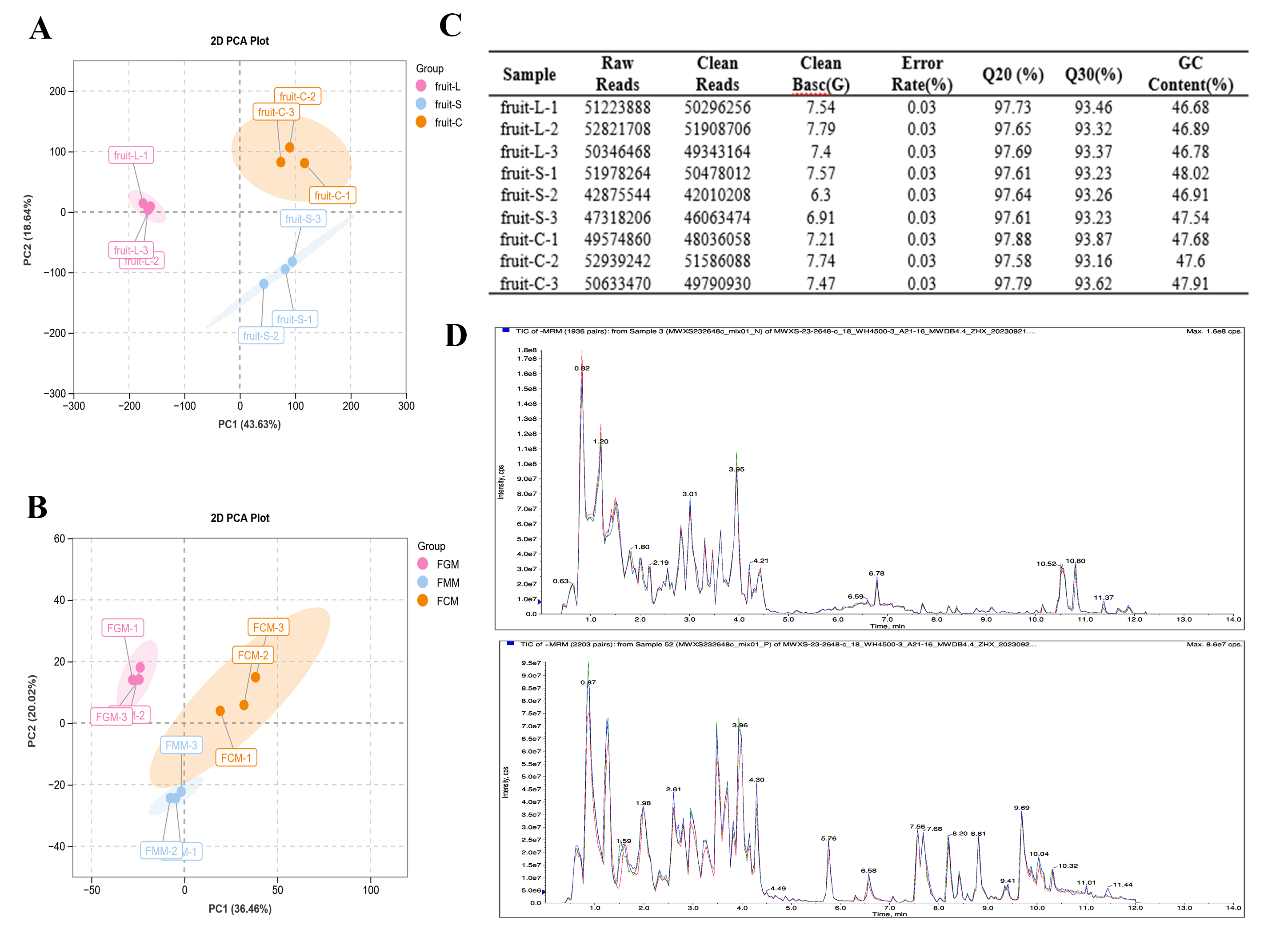


**Supplementary Figure 1.** Data quality control (A) Transcriptome sample principal component analysis 2D score plot (B) Metabolome sample principal component analysis 2D score plot (C) Quality control of *Artocarpus nanchuanensis* transcriptome data (D) Mixed sample quality spectrum analysis total ion flow map (TIC) overlap diagram

**
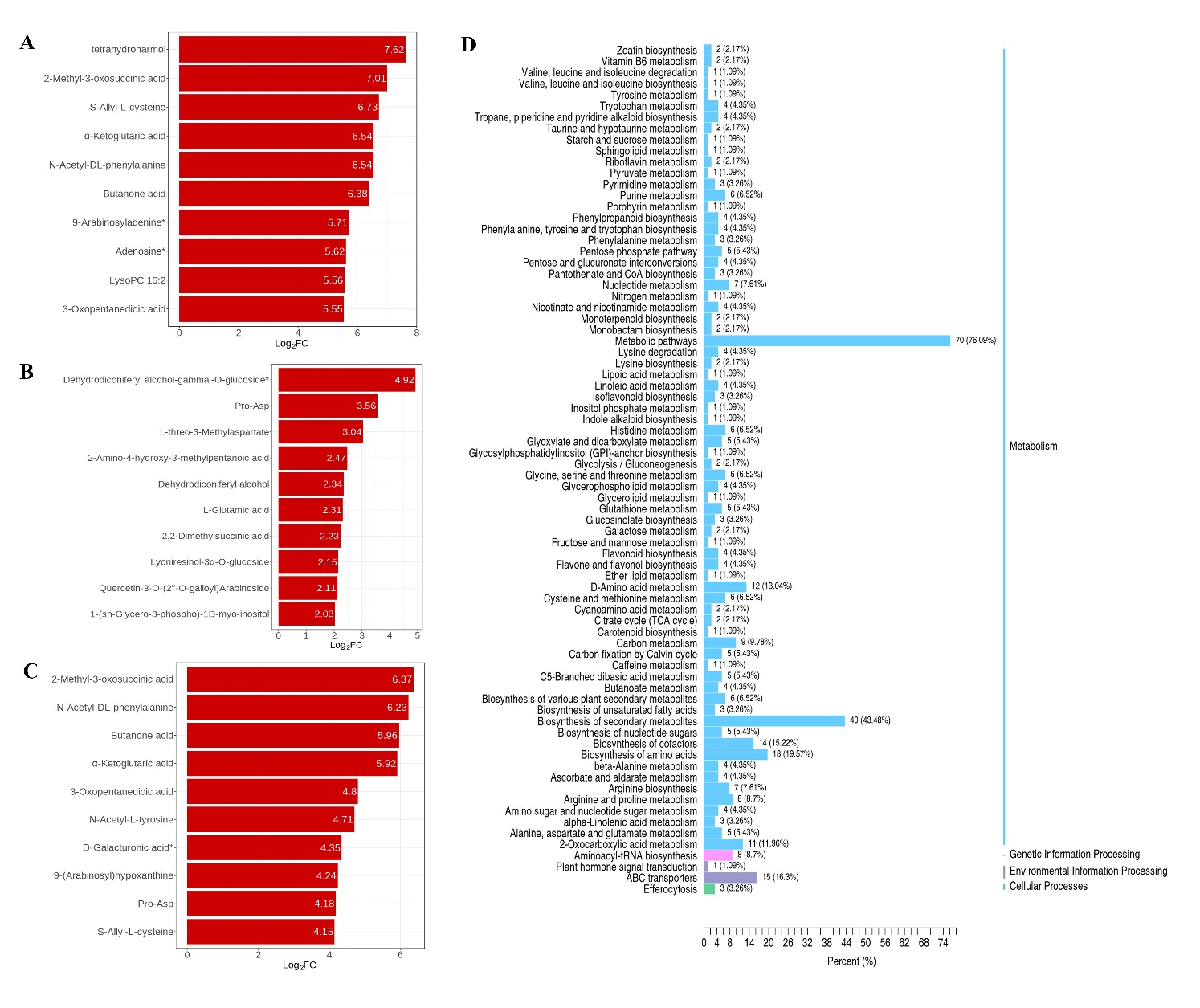
**

**Supplementary Figure 2.** (A) Dynamic distribution of the top 10 significantly upregulated metabolites in FCM vs FGM. (B) Dynamic distribution of the top 10 significantly upregulated metabolites in FMM vs FCM. (C) Dynamic distribution of the top 10 significantly upregulated metabolites in FMM vs FGM. (D) KEGG classification of 407 overlapping metabolites.

**
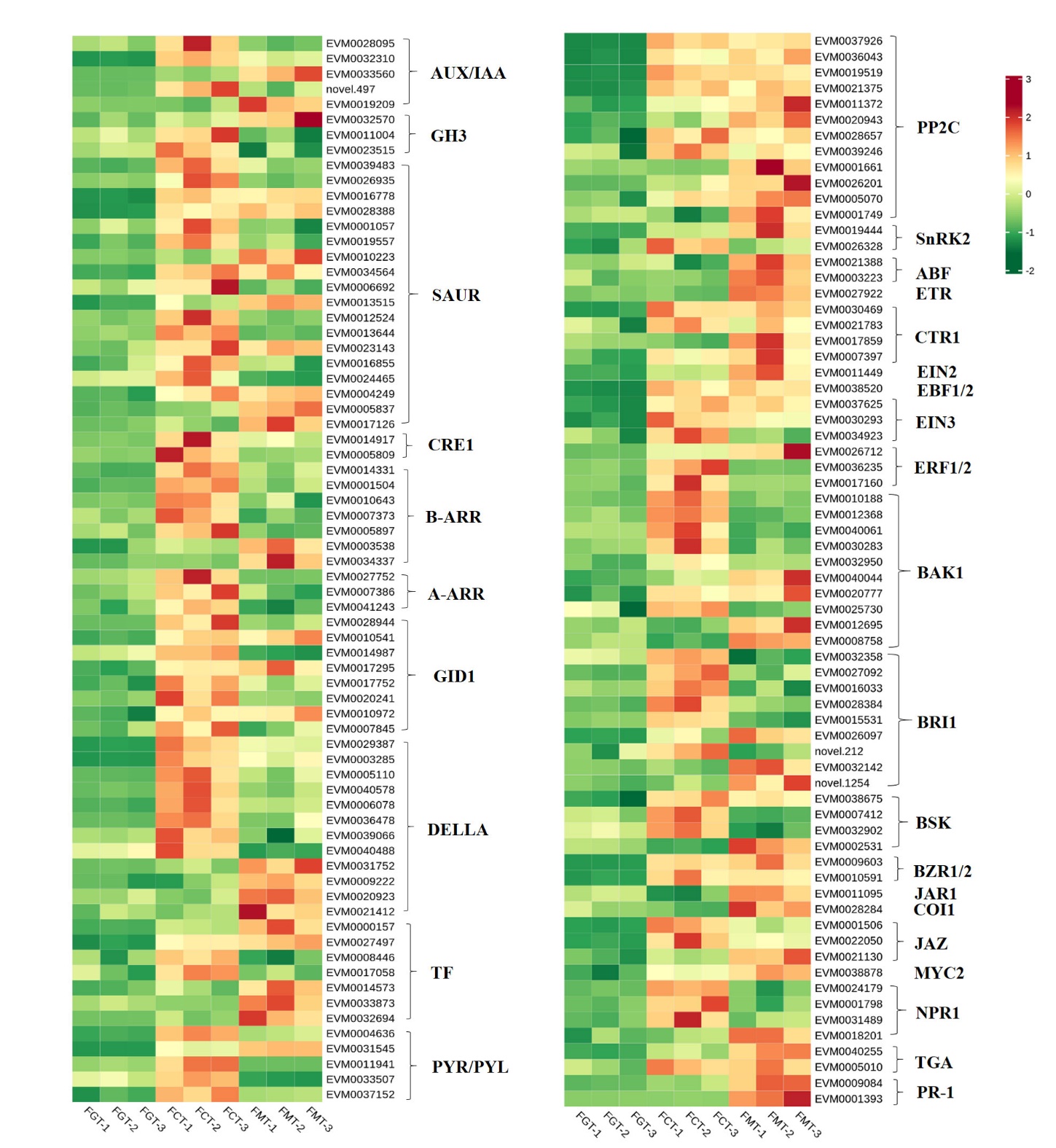
**

**Supplementary Figure 3.** Expression patterns of 138 DEGs associated with the plant hormone signal transduction pathway.

**
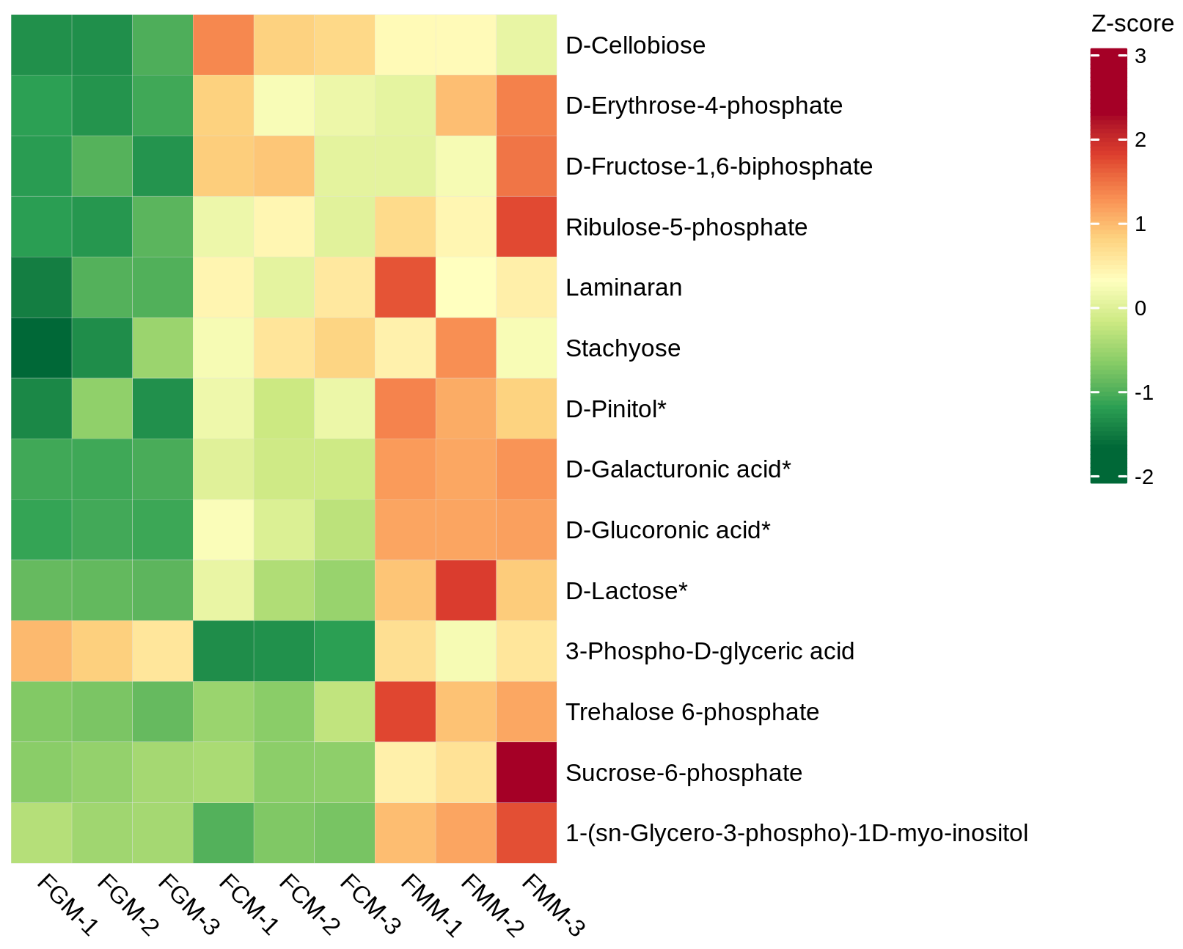
**

**Supplementary Figure 4.** Heatmap of 14 overlapping saccharides metabolites identified among FCM vs FGM, FMM vs FCM and FMM vs FGM comparisons.

**
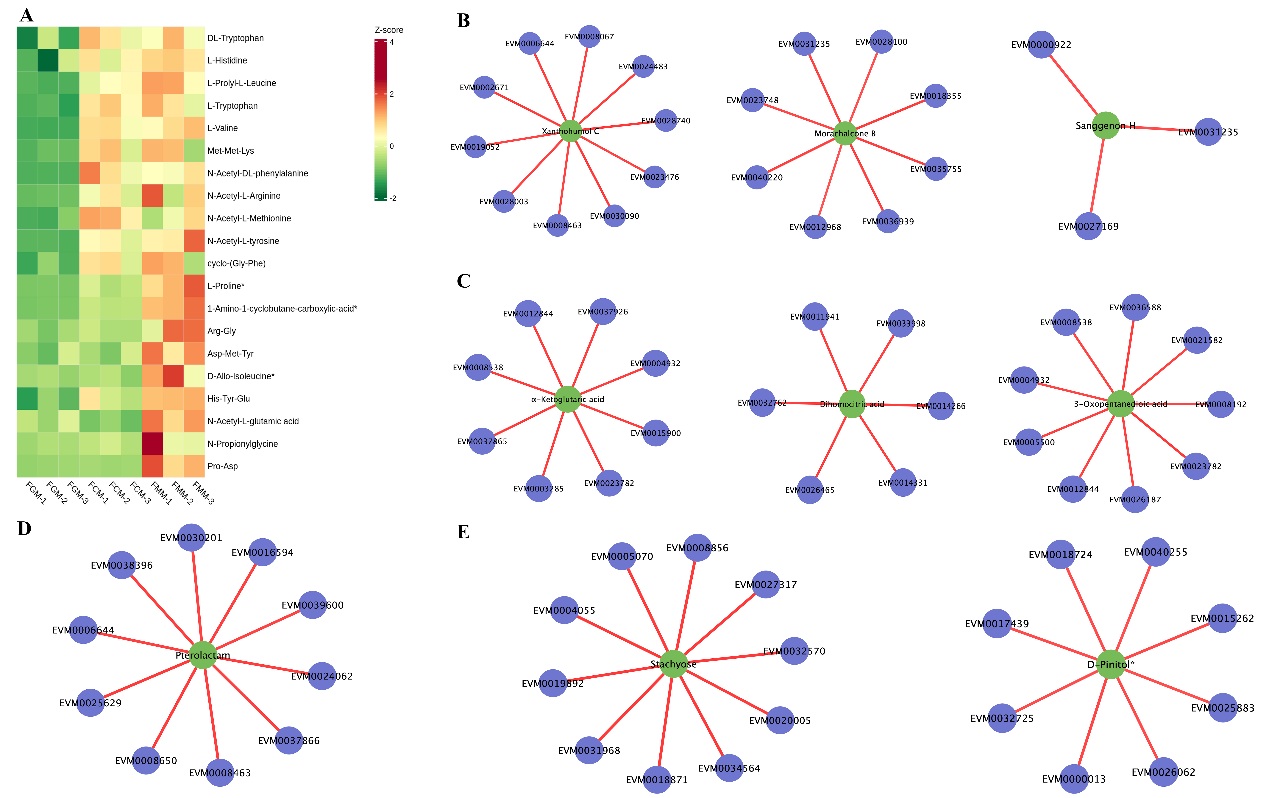
**

**Supplementary Figure 5.** (A) Heatmaps displaying expression patterns of 20 overlapping differential accumulated amino acids among 3 comparison groups. (B) The correlation network diagram for flavonoids metabolites and some DEGs, including Xanthohumol C, Morachalcone B, and Sanggenon H. *EVM0018355*, *EVM0035755*, *EVM0036939* and *EVM0040220*, *major allergen Pru av 1*; *EVM0023748*, *galactinol-sucrose galactosyltransferase 5*; *EVM0027169*, *phosphatase 2C*; *EVM0028100*, *UDP-glycosyltransferase*; *EVM0028740*, *EVM0023476*, *EVM0012968*, *E3 ubiquitin-protein ligase*; *EVM0030090*, *SAUR 18*; *EVM0002671*, *EVM0006644*, *ABC transporter G family member 11*; *EVM0024483*, *early responsive to dehydration 15*; *EVM0008067*, *receptor-like protein kinase Feronia*; *EVM0028003*, *bidirectional sugar transporter SWEET17*; *EVM0008463*, *IAA21*; *EVM0019052*, *WRKY*; *EVM0031235*, *MYB*; *EVM0000922*, *C2-domain ABA-related 5*. (C) The correlation network diagram for organic acid metabolites and some DEGs, including Dihomocitric acid, 3-Oxopentanedioic acid, and α-Ketoglutaric acid. *EVM0011941*, *ABA receptor PYL4*; *EVM0032762, WRKY*; *EVM0026465*, *gibberellin 2-oxidase 4*; *EVM0014331*, *GARP-G2-like*; *EVM0033998*, *MYB*; *EVM0008538*, *E2F*; *EVM0036588*, *EVM0008192*, *E3 ubiquitin-protein ligase*; *EVM0037926*, *phosphatase 2C*; *EVM0014266*, *xyloglucan endotransglucosylase/hydrolase 2*; *EVM0005500*, *cycloartenol-C-24-methyltransferase;* *EVM0026187*, *HB-HD-ZIP*; *EVM0021582*, *C2H2*; *EVM0023782*, *disease resistance protein RPM1*; *EVM0012844*, *MYB*; *EVM0004932*, *heat stress transcription factor, HSF*; *EVM0015900, Ubiquitin-conjugating enzyme E2;EVM0003285,* *DELLA protein GAI (A); EVM0037865*, *disease resistance protein RPM1*. (D) The correlation network diagram for alkaloid pterolactam and some DEGs. *EVM0006644*, *ABC transporter G family member*; *EVM0030201*, *TMV resistance protein N-like*; *EVM0008463*, *IAA21*; *EVM0025629*, *EVM0037866*, *Constans-like*; *EVM0008650*, *plant cadmium resistance 3-like*; *EVM0024062*, *EVM0038396,* *ABC transporter F family member*; *EVM0016594*, *methyltransferase*; *EVM0039600*, *bHLH*. (E) The correlation network diagram for saccharides metabolites and some DEGs, including stachyose and D-Pinitol*. *EVM0004055*, *UDP-glycosyltransferase*; *EVM0032570*, *indole-3-acetic acid-amido synthetase*; *EVM0034564*, *auxin-responsive protein* *SAUR71*; *EVM0019892*, *phenylalanine ammonia lyase*; *EVM0018871*, *major allergen Pru av 1*; *EVM0031968*, *MYB*; *EVM0027317*, *dehydrin Rab15*; *EVM0008856*, *selenium-binding protein 3*; *EVM0020005*, *ERF*; *EVM0005070*, *phosphatase 2C*; *EVM0000013*, *F-box domain containing protein*; *EVM0015262*, *CYP450*; *EVM0017439*, *heat shock protein*; *EVM0026062*, *E3 ubiquitin-protein ligase DIS1*; *EVM0032725*, *carbonic anhydrase 2*; *EVM0018724*, *AT-hook motif nuclear-localized protein 28*; *EVM0025883*, *patatin-like protein 2*; *EVM0040255*, *bZIP*.


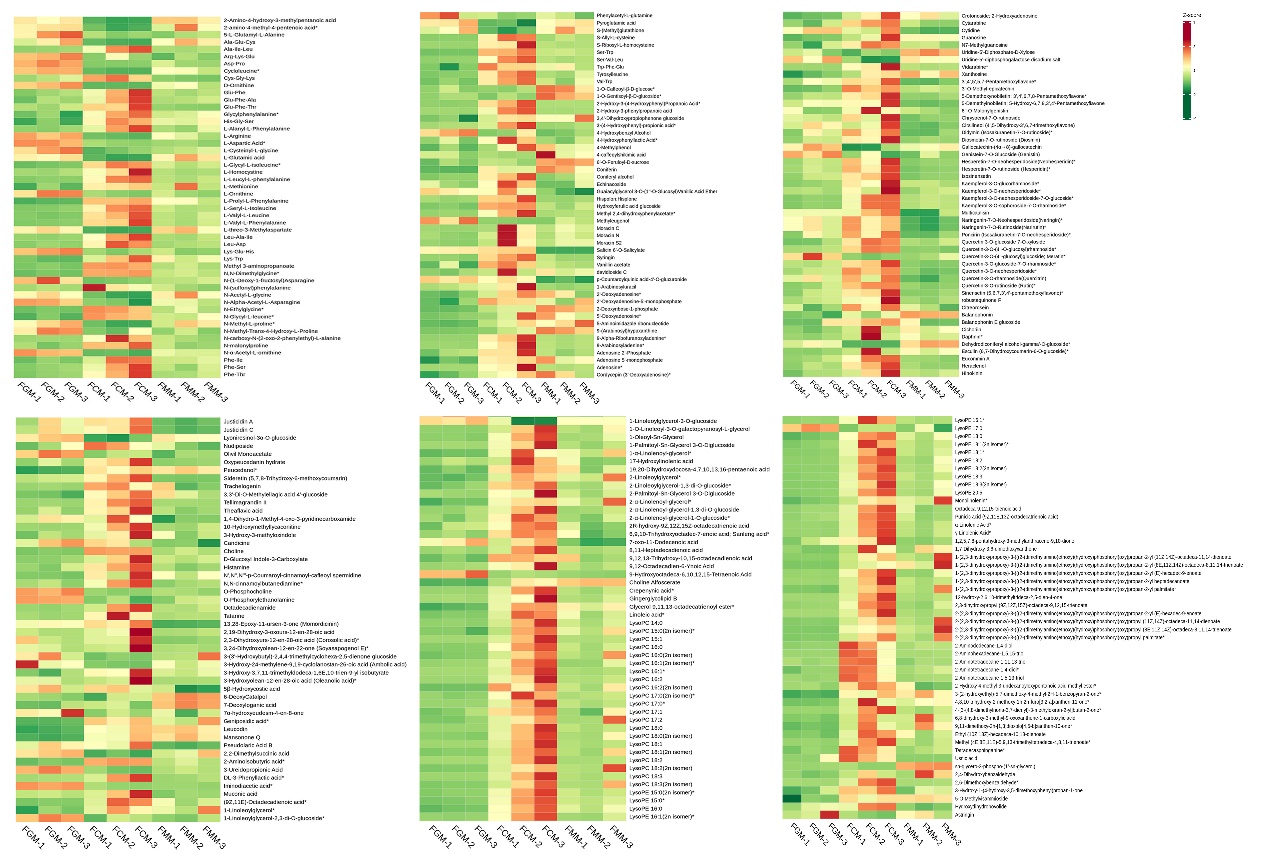


**Supplementary Figure 6.** Heatmap illustrating the dynamic changes of shared DAMs among FCM vs FGM, FMM vs FCM and FMM vs FGM comparisons.

Supplementary Table 1 The relative abundance of key metabolites across the three developmental stages.

| Compounds | FGM-1 | FGM-2 | FGM-3 | FCM-1 | FCM-2 | FCM-3 | FMM-1 | FMM-2 | FMM-3 |
| --- | --- | --- | --- | --- | --- | --- | --- | --- | --- |
| Laminaran | 35270.4 | 56843.1 | 56145.4 | 118390.1 | 102036.7 | 124603.1 | 173571.3 | 113391.8 | 121179.5 |
| D-Lactose* | 706961.3 | 680429.8 | 593277.1 | 2747476.3 | 1742584 | 1400254 | 4465535 | 6404584 | 4357017 |
| D-Cellobiose | 103019.7 | 101667.8 | 147280.6 | 492214.1 | 414894.5 | 404483.5 | 350586 | 319038.4 | 294327.2 |
| α-Ketoglutaric acid | 1009683 | 936645 | 999657.6 | 96977261.2 | 92677865 | 85050981 | 55041163 | 68570279 | 54698722 |
| 3-Oxopentanedioic acid | 18839.4 | 72522.1 | 21358.4 | 1712715.4 | 1556394 | 2001006 | 914001.5 | 1416845 | 815866.8 |
| Dihomocitric acid | 104093 | 119716.5 | 141691.4 | 3763514.6 | 5553576 | 6178765 | 793942.1 | 439566.8 | 1284692 |
| Anthriscifolcine A | 1017520 | 1197269 | 1190459 | 15778874.7 | 17042297 | 25399025 | 3698931 | 2639389 | 8478324 |
| Pterolactam | 664910.2 | 761817.7 | 744733.6 | 2770067.6 | 1929343 | 2008630 | 4877788 | 5034159 | 6713908 |
| tetrahydroharmol | 28953.6 | 43400.7 | 31941.9 | 4893442.4 | 5468553 | 10190018 | 363732.6 | 202247.6 | 625039 |
| Luteolin | 116950.4 | 135496.2 | 90663.4 | 376047.8 | 851599.8 | 506866.8 | 655600.8 | 866898 | 586172.2 |
| Phloretin | 10003.5 | 4191.272 | 2400.398 | 12783.76 | 15442.3 | 13728.55 | 24697.1 | 20001.2 | 17289.9 |
| Xanthohumol C | 1198.368 | 2733.61 | 2827.723 | 23811.525 | 25231.8 | 60860.7 | 5648.863 | 3165.708 | 7663.551 |
